# Balancing a genetic toggle switch by real-time feedback control and periodic forcing

**DOI:** 10.1038/s41467-017-01498-0

**Published:** 2017-11-17

**Authors:** Jean-Baptiste Lugagne, Sebastián Sosa Carrillo, Melanie Kirch, Agnes Köhler, Gregory Batt, Pascal Hersen

**Affiliations:** 1 Laboratoire Matière et Systèmes Complexes, UMR 7057 CNRS & Université Paris Diderot, 10 rue Alice Domon et Léonie Duquet, 75013 Paris, France; 2 Inria Saclay–Ile-de-France and Université Paris Saclay, 1 rue Honoré d’Estienne d’Orves, Bâtiment Alan Turing, Campus de l’Ecole Polytechnique, 91120 Palaiseau, France; 3 Institut Pasteur, 25-28 Rue du Docteur Roux, 75015 Paris, France

## Abstract

Cybergenetics is a novel field of research aiming at remotely pilot cellular processes in real-time with to leverage the biotechnological potential of synthetic biology. Yet, the control of only a small number of genetic circuits has been tested so far. Here we investigate the control of multistable gene regulatory networks, which are ubiquitously found in nature and play critical roles in cell differentiation and decision-making. Using an in silico feedback control loop, we demonstrate that a bistable genetic toggle switch can be dynamically maintained near its unstable equilibrium position for extended periods of time. Importantly, we show that a direct method based on dual periodic forcing is sufficient to simultaneously maintain many cells in this undecided state. These findings pave the way for the control of more complex cell decision-making systems at both the single cell and the population levels, with vast fundamental and biotechnological applications.

## Abstract (web summary)

Cybergenetics aims to monitor and regulate cellular processes in real-time using computer monitoring and feedback of biological readouts. Here the authors use a feedback loop and periodic forcing to maintain cells with a bistable synthetic circuit near its unstable state.

## Introduction

Only recently has feedback control been applied to take control of simple biological functions^1–8^. Typically, a control algorithm is used to decide how to stimulate the expression of a fluorescent reporter gene (e.g., by controlling the concentration of a drug in the cellular environment) based on the difference between the actual and target fluorescence levels. Although such methods are directly relevant to physical systems, they are very difficult to apply in a biological context–at least in part due to the stochastic nature of gene expression, un-modeled dynamics and the limited number of methods with which to interact with cells. Thus, to date, feedback control has only been applied to simple objectives such as controlling the level of expression of a reporter gene at a constant level over long periods of time^1,2^. However, to demonstrate the potential of computer-based feedback control of cellular processes for systems and synthetic biology, control methods need to be validated using complex, hard-to-control biological systems. Stabilizing a pendulum in its upward unstable equilibrium position is a classic benchmark in physical control theory. By analogy, successfully driving and maintaining a bistable genetic circuit to its unstable equilibrium state would represent a milestone in the development of real-time computer-based feedback control of gene regulatory networks. Indeed, as bistable circuits display hysteresis, they raise specific control challenges and are ideal systems to demonstrate the potential of cybergenetics^9^.

**Fig. 1:**
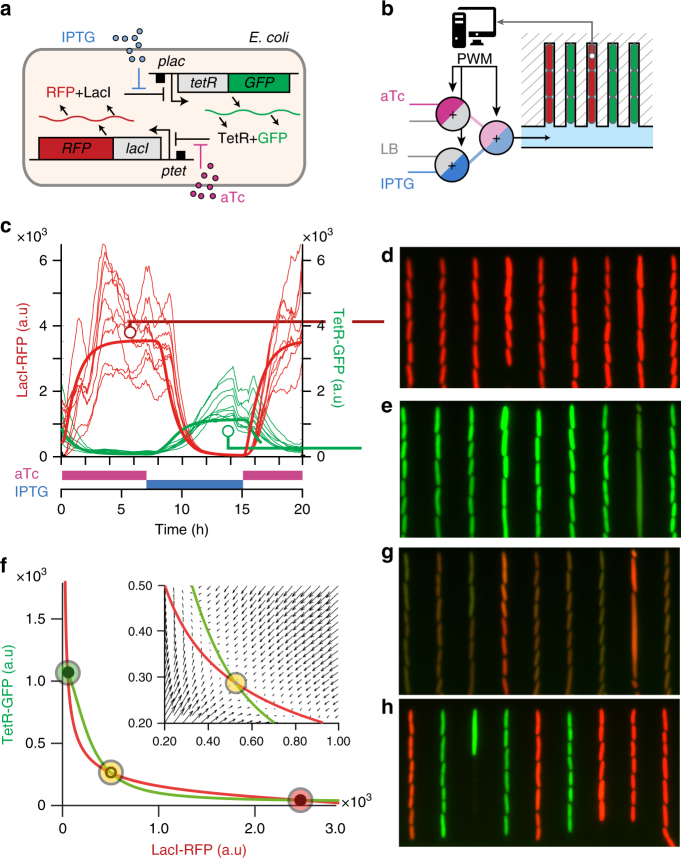
Construction and characterization of a genetic toggle switch that displays bistable behavior. **a** The genetic toggle switch is composed of two mutually repressing branches, the strength of each can be tuned by addition of two chemicals (aTc or IPTG) to the cellular environment. The levels of the two repressors (LacI and TetR) can be observed using two fluorescent reporters (mKate2 and mEGFP). **b** *E. coli* cells are forced to grow in parallel lines in a microfluidic device to facilitate cell segmentation and tracking. Three valves used in pulse-width-modulation (PWM) allow the modulation of the concentrations of aTc and IPTG in real-time (Supplementary Fig. 2). **c** To calibrate a model of the toggle switch (see “Methods” section), the single-cell responses of red (LacI) and green (TetR) fluorescence were measured as a function of step changes in aTc and IPTG concentrations (*n*=9, see also Supplementary Fig. 3). Model outputs for averaged LacI and TetR levels are represented by the thick red and green lines. As expected for a toggle switch circuit, in presence of excess aTc, all cells are in a red-dominant (LacI) state (**d**), while all cells switch to a green-dominant (TetR) state in the presence of excess IPTG (**e**). **f** LacI (red) and TetR (green) nullclines of the fitted model under reference conditions (aTc = 20 ng ml^−1^; IPTG = 0.25 mM). The state space presents two stable equilibrium points (red and green circles) and one unstable equilibrium point (yellow circle). The inset shows the vector field close to the unstable point. Stability analysis of the model indicated that for a large region of the state space, in which protein levels are comparable, no static combinations of aTc and IPTG concentrations could be used to maintain a cell in that region (see Supplementary Fig. 6). Experimentally, while cells transiently expressing both green and red fluorescence could be observed **g**, the cells eventually committed to one of the two stable states **h** as expected for a bistable toggle switch, illustrating that dynamic control is required to maintain cells away from their two stable attractors

The toggle switch is a well-characterized bistable biological circuit. The first synthetic toggle switch was established by Gardner and Collins^10^ in *Escherichia coli*. Briefly, this system consists of two genes, *lacI* and *tetR*, that mutually repress each other (Fig. 1a). The system displays two stable equilibrium states in which either of the two gene products represses the expression of the other gene. Once cells are committed to an equilibrium state, they remain in that state even in presence of small perturbations. Therefore, the toggle switch has been proposed as a canonical circuit for understanding cell decision making and cell fate differentiation^10–14^. The repression of *lacI* and *tetR* on their promoters can be tuned by addition of the diffusible molecules, isopropyl-β-D-thiogalactopyranoside (IPTG) and anhydrotetracycline (aTc), respectively. Hence, it is possible to interact with and switch the circuit from one stable equilibrium state to the other by addition of aTc or IPTG. In theory, the genetic toggle switch has another equilibrium state in which both genes are expressed at comparable (low) levels. However, this equilibrium is unstable, and lies on a curve separating the two basins of attraction of the stable equilibrium states called the “separatrix”. In particular Wu et al. recently showed that cells starting from a null expression of both LacI and TetR are stochastically driven towards one of the two attractors^12^. More generally, cells cannot stay near the separatrix for long, since (stochastic) perturbations eventually push the cells into one of the two basins of attraction^12,15–18^. Therefore, and as for the case of unstable mechanical systems, a dynamic control strategy is required to maintain cells away from the two attractors. However, real-time control of cellular processes is a recent, challenging method. This explains why maintaining cells near the unstable state and away from the two stable attractors has never been achieved experimentally. However, it is extremely desirable to drive and maintain cells in such unstable equilibrium states, since they are central for cell decision-making and fate commitment. Here we demonstrate experimentally that a cellular toggle switch can be stabilized close to the attractor basin boundaries by dynamically varying the concentrations of IPTG and aTc.

## Results

### Bistability of the genetic toggle switch

A genetic toggle switch was built by constructing a plasmid in which LacI and TetR are transcriptionally fused to the fluorescent reporters *mKate2* (RFP) and *mEGFP* (GFP; Fig. 1a and Supplementary Fig. 1). The *E. coli* cells were transformed with the plasmid and cultured in a microfluidic device in which the concentrations of aTc and IPTG could be varied dynamically (Fig. 1b and Supplementary Fig. 2). The state of the toggle switch was observed at the single-cell level over extended periods of time by monitoring the levels of GFP and RFP via fluorescence microscopy (see “Methods” section). The capacity of the cells to switch to a RFP-dominant state (or GFP-dominant state) upon addition of aTc (or IPTG, respectively) and their ability to retain this state after washout of the inducer (Fig. 1c–e, Supplementary Fig. 3) were tested. Furthermore, the basal concentrations of aTc ($u^0_{\rm aTc}=$20 ng mL^−1^) and IPTG ($u^0_{\rm IPTG} =$0.25 mM) were adjusted to achieve a robust, bistable behavior (Fig. 1c–e). Under these conditions, all cells eventually moved to either one of the two stable states (Fig. 1f–h), as expected for a bistable circuit.

### Quantitative model of the toggle switch circuit

To estimate the location of the stable and unstable equilibrium points in the state space (LacI-RFP, TetR-GFP), we developed a deterministic, Hill-type model of the toggle switch (see “Methods” section), generated calibration data in time-varying environments of aTc and IPTG (Fig. 1c and Supplementary Fig. 3), and fitted the model to these data using literature information (Supplementary Table 1) and a global optimization tool, Covariance Matrix Adaptation Evolution Strategy (CMAES, “Methods” section, Supplementary Fig. 3). The model is based on pseudo-reactions that represents: transcription:

$${\emptyset \mathop { \to }\limits^{f_{\rm{L}}^{\rm m}\left( {{\rm{TetR}},{\rm{aTc}}} \right)} {\rm{mRNA}}_{\rm{L}}} \,{\rm and} \,\emptyset \mathop { \to }\limits^{f_{\rm{T}}^{\rm m}\left( {{\rm{LacI}},{\rm{IPTG}}} \right)} {\rm{mRN}}{{\rm{A}}_{\rm{T}}}$$

translation:

$${\rm{mRN}}{{\rm{A}}_{\rm{L}}}\mathop { \to }\limits^{k_{\rm{L}}^{\rm p}} {\rm{mRN}}{{\rm{A}}_{\rm{L}}} + {\rm{LacI}} \,{\rm and}\, {\rm{mRN}}{{\rm{A}}_{\rm{T}}}\mathop { \to }\limits^{k_{\rm T}^{\rm p}} {\rm{mRN}}{{\rm{A}}_{\rm{T}}} + \left.{\rm{TetR}} \right),$$

and degradation/dilution due to growth:

$${\rm{mRN}}{{\rm{A}}_{\rm{L}}}\mathop { \to }\limits^{g_{\rm L}^{\rm m}} \emptyset, {\rm{mRN}}{{\rm{A}}_{\rm{T}}}\mathop { \to }\limits^{g_{\rm{T}}^{\rm m}} \emptyset, {\rm{LacI}}\mathop { \to }\limits^{g_{\rm{L}}^{\rm p}} \emptyset \, {\rm and} \, {{\rm TetR}}\mathop { \to }\limits^{g_{\rm T}^{\rm p}} \emptyset.$$

$f_{\rm L}^{\rm m}\left( {{{TetR}},aTc} \right) = k_{\rm{L}}^{{\rm m}0} + k_{\rm{L}}^{\rm m} \cdot {h^ - }\left( {{\rm{TetR}} \cdot {h^ - }\left( {aTc,{\theta _{{\rm{aTc}}}},{\eta _{{\rm{aTc}}}}} \right),{\theta _{{\rm{TetR}}}},{\eta _{{\rm{TetR}}}}} \right)$ and $f_{\rm{L}}^{\rm m}\left( {{{LacI}},{{IPTG}}} \right) = k_{\rm{T}}^{{\rm m}0} + k_{\rm{T}}^{\rm m} \cdot {h^ - }\left( {{{LacI}} \cdot {h^ - }\left( {{{IPTG}},{\theta _{{\rm{IPTG}}}},{\eta _{{\rm{IPTG}}}}} \right),{\theta _{{\rm{LacI}}}},{\eta _{{\rm{LacI}}}}} \right)$ are gene regulation functions, $k_{{\rm{L}}/{\rm{T}}}^{{\rm m}0}$, $k_{{\rm{L}}/{\rm{T}}}^{\rm m}$, $k_{{\rm{L/T}}}^{\rm p}$, $g_{{\rm{L}}/{\rm{T}}}^{\rm m}$, and $g_{{\rm{L}}/{\rm{T}}}^{\rm p}$ leakage transcription, transcription, translation, mRNA degradation, and protein degradation rate parameters, and ${h^ - }\left( {{\it{x}},\theta ,\eta } \right) = 1/\left( {1 + {{\left( {x/\theta } \right)}^\eta }} \right)$ being the decreasing Hill function. Threshold parameters of the Hill function are expressed in arbitrary fluorescence units (a.u.) for proteins (*θ*_LacI_ and *θ*_TetR_),  ng mL^−1^ for aTc (*θ*_aTc_), and mM for IPTG (*θ*_IPTG_). We assumed the transcription rate is a decreasing Hill function of the free repressor, the latter being the product of the total repressor (TetR or LacI) and a decreasing Hill function of its allosteric regulator (aTc or IPTG, respectively). The rates of translation and mRNA and protein degradation/dilution due to growth are assumed to be proportional to the reactants. Depending on the problem, a deterministic or stochastic interpretation of this set of pseudo-reactions was employed. The stochastic interpretation results in a continuous time Markov chain model (see “Methods” section). Mathematically, the deterministic interpretation results in a set of four ordinary differential equations (ODEs):

$${\frac{{{\rm{d}}\,{mRN}{{{A}}_{{\rm{LacI}}}}}}{{{\rm{d}}t}} = \kappa _{\rm{L}}^{{\rm m}0} + \frac{{\kappa _L^m}}{{1 + {{\left( {\frac{{{{TetR}}}}{{{\theta _{{\rm{TetR}}}}}} \times \frac{1}{{1 + {{\left( {{ aTC}/{\theta _{\rm aTC}}} \right)}^{{\eta _{\rm aTC}}}}}}} \right)}^{{\eta _{\rm TetR}}}}}} - g_{\rm L}^{\rm m} \times {mRN}{{{A}}_{{\rm{LacI}}}}}$$

$${\frac{{{\rm{d}}\,{mRN}{{A}_{{\rm{TetR}}}}}}{{{\rm{d}}t}} = \kappa _{\rm T}^{{\rm m}0} + \frac{{\kappa _T^m}}{{1 + {{\left( {\frac{{{{LacI}}}}{{{\theta _{{\rm{LacI}}}}}} \times \frac{1}{{1 + {{\left( {{{IPTG}}/{\theta _{{\rm{IPTG}}}}} \right)}^{{\eta _{\rm IPTG}}}}}}} \right)}^{{\eta _{\rm LacI}}}}}} - g_{\rm T}^{\rm m} \times {mRN}{{A}_{{\rm{TetR}}}}}$$

$$\frac{{{\rm{d}}\,{{LacI}}}}{{{\rm{d}}t}} = \kappa _{\rm L}^{\rm p} \times {{mRN}}{{{A}}_{{\rm{LacI}}}} - g_{\rm L}^{\rm p} \times {{LacI}}$$

$$\frac{{{{{\rm{d}}TetR}}}}{{{\rm{d}}t}} = \kappa _{\rm T}^{\rm p} \times {{mRN}}{{{A}}_{{\rm{TetR}}}} - g_{\rm T}^{\rm p} \times {TetR}$$

To obtain a good fit to calibration, and subsequently, the control data, we added a model to account for inducer exchange in and out of the cell (“Methods” section).

The model was essential to predict the phase portrait of the toggle switch–which cannot be obtained experimentally–and the theoretical positions of the stable and unstable equilibrium points (Fig. 1f). The model consistently predicted the toggle switch to be bistable under the reference conditions and the corresponding unstable equilibrium state to be found at relatively low levels for both proteins (Fig. 1f and Supplementary Figs. 4–6). Due to phenotypic heterogeneity, one should not expect two different cells to have the exact same bistable dynamics and equilibrium locations. Yet, the fitted model was instrumental to explore the relevance of different control methods and guide the design of experiments. Note that phenotypic heterogeneity is not a problem for controlling a single cell but may limit the efficiency of a dynamic control applied to several cells at once.

### Cells can be dynamically maintained in the unstable area

**Fig. 2:**
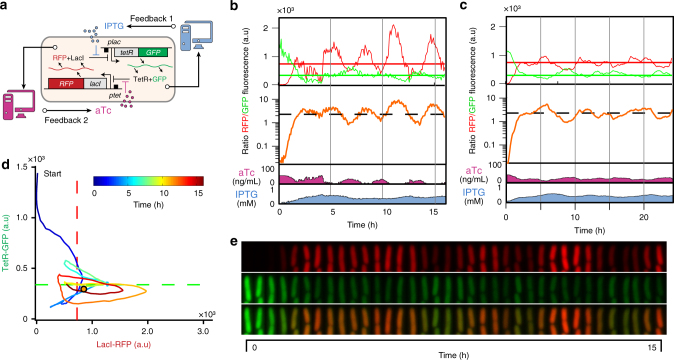
Cells can be maintained close to the target control point using a dual PI controller. **a**. A dual controller, in which aTc and IPTG levels were changed independently from one another, was used to reach the respective target levels of RFP and GFP. **b**. Control experiment in which the dual PI controller is applied to drive an *E. coli* cell. Temporal evolutions of the red (LacI-RFP) and green (TetR-GFP) fluorescence levels for the controlled cell are represented (top). The horizontal red and green lines are the target values of both controller branches. The controlled protein levels, although displaying marked oscillations, remains close to their target values. The ratio (orange, middle) of red and green fluorescence shows that protein expression levels remain comparable over several hours. The concentrations of the inducers applied by the dual PI controller are represented as a function of time (bottom). The parameters of the PI dual controller are ${\rm K}_{\rm P}^{\rm L} = 3.3\,10^{-2},{\rm K}_{\rm I}^{\rm L} = 1.32\,10^{-4}\,{\rm s}^{-1}$, ${\rm K}_{\rm P}^{\rm T} = 1.65\,10^{-2},{\rm K}_{\rm I}^{\rm T} = 4.6\,10^{-4}\,{\rm s}^{-1}$ (see “Methods” section). **c** In silico control experiment in which the dual PI controller is applied to drive a stochastic version of the toggle switch model. The parameters of the PI dual controller are as in (**b**). **d** Smoothed trajectory of the experimentally controlled cell in the (LacI-RFP, TetR-GFP) state space. The controlled cell remains close to the target control point and at some distance from the attractive states, where LacI or TetR dominate. **e**. Fluorescence images (RFP, GFP, merge) of cells under remote control shown in **d**, **c** (cell size  ~ 1 µm). The controlled cells express comparable levels of both RFP and GFP. See Fig. 3 and Supplementary Fig. 7 for additional single-cell control experiments

**Fig. 3:**
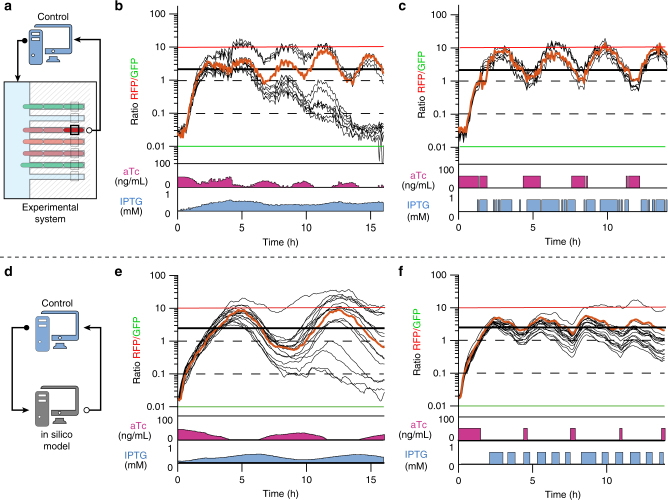
Additional control experiments show that single cells can be controlled with PI and Bang-Bang controllers. **a** Experimental control principle. The controller uses the fluorescence measurements for a single cell (the controlled cell) to compute control inputs (aTc and IPTG levels). Yet, all cells in the field of view were imaged during the process. **b** Ratio of RFP (LacI) and GFP (TetR) levels for the controlled (orange) and in the field of view (black, *n* = 11) cells for the control experiment shown in Fig. 2c. Some cells escaped to a GFP-dominant state, while others remained close to the control target. **c** Using a bang-bang control strategy (on/off control) led to an equally good result for the controlled cell. Surprisingly, in this case, all cells (*n* = 8) faithfully followed the controlled cell. **d** In silico control principle. **e**, **f**. Similar results to those in **b**, **c** were observed in the in silico control experiments. Trajectories (*n* = 16) were obtained by simulating the behavior of cells placed in the same dynamic environment than that of the controlled cell

By analogy with the inverted pendulum control problem, our first goal was to maintain a single cell close to the predicted unstable equilibrium point by dynamically adjusting the concentrations of aTc and IPTG. Real-time changes in the concentrations of inducers were computed using an in silico feedback controller based on the levels of the fluorescent reporters GFP and RFP (Fig. 2a). Initially, two proportional-integral (PI) controllers were employed to regulate the expression levels of *lacI* and *tetR*. More precisely, the first (or second) controller adjusts the concentration of aTc (or IPTG) so as to minimize the deviations of the LacI-RFP (or TetR-GFP) level from its target level, as shown in Fig. 2a. Note that, due to the specific topology of the toggle switch, the two controllers have adverse effects on each other: each controller aims to maintain the concentration of the controlled protein close to a (relatively low) target level, and this weakens the effect of the addition or removal of the inducer used by the other controller. This control strategy was experimentally tested on a randomly-chosen individual cell. As shown in Fig. 2b–d and Supplementary Movie 1, the feedback control algorithm succeeded in maintaining the controlled cell close to the target control point, effectively preventing the cell from committing to either one of the two stable states. In the state space, each controlled cell did not remain at a fixed position, but followed a large, noisy trajectory around the target point (Fig. 2d, Supplementary Movie 1). Comparable results were obtained in silico by using the same PI controllers to drive a simulated cell whose behavior was given by a stochastic interpretation of our model of the toggle switch (“Methods” section and Fig. 2c). Using a PI controller requires to use a complex mixing device upstream of the microfluidic channels to vary the concentrations of both aTc and IPTG in real time. We thus tried a simpler control strategy (Bang-Bang controller) in which each controller applies a minimal or maximal concentration of inducer, depending on the sign of the difference between the target and observed fluorescence values (Fig. 3c, f, and Supplementary Movie 2). This strategy gave excellent control results while being technically less challenging. Taken together, the controlled cells consistently remained away from the stable states as long as the control method was active for all experimental and numerical control experiments (Figs. 2b, e and 3b, c, e, f, and Supplementary Fig. 7). As such, we demonstrated that it is indeed possible to use dynamic control to force a single cell to remain in an undecided state that is near the unstable equilibrium position. This is an important achievement and a novel method for the fundamental study of decision making in multistable gene circuits. But, the control efficiency comes at a price: only one single cell can be controlled at a time. This in turn limits the use of real-time control methods for biotechnological applications.

### Periodic forcing can simultaneously stabilize several cells

**Fig. 4:**
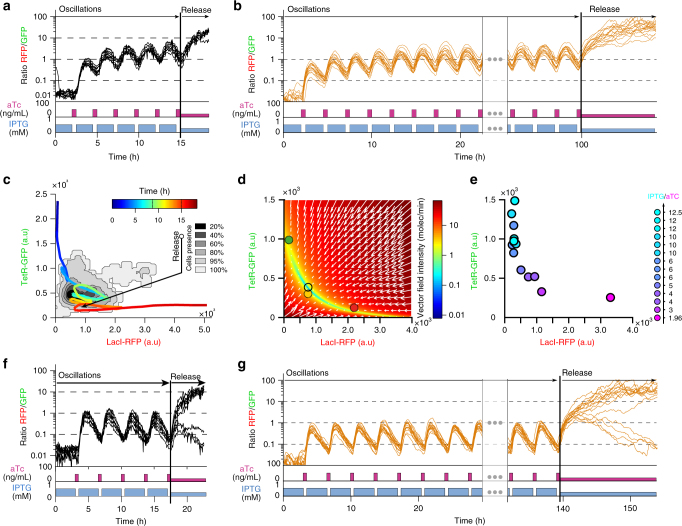
A cell population can be maintained in a state of balanced expression using periodic stimulations. **a**. Ratio of LacI-RFP and TetR-GFP levels for observed cells (*n* = 8). The concentrations of the inducers were varied periodically (120 min of 0.5 mM IPTG, 30 min of 50 ng ml^−1^ aTc). In such dynamic conditions, the cells were kept in a state of balanced expression. As soon as the periodic stimulation was stopped, with aTc and IPTG set back to their reference levels, the cells were attracted to the RFP-dominant state. **b** In silico experiment, in which periodic stimulation was applied as in **a** to a simulated population of cells (n = 16) implementing a stochastic version of the toggle switch model and showing a similar behavior. **c** Experimental trajectory of one of the cells shown in **a** in the (LacI-RFP; TetR-GFP) state space. Cell probability of presence is shown for the time window (9 h < Time < 15 h). **d** Using the model, the vector field in the state space averaged over time during an entire period of stimulation can be computed. The vector field displays a single null point that acts like an effective global attractor (black circle). **e** The global localization of the cells in the state space during the oscillatory regime depends on the relative amounts of inducers. The average position of the cell population is represented for 14 different periodic stimulation experiments that differed by the total amount of IPTG and/or aTc used. The experiment depicted in (**a**, **c** has a ratio of 4 (violet circle). **f** Experimental periodic stabilization for a different stimulation frequency (180 min of 50 mM IPTG, 30 min of 50 ng ml^−1^ aTc, *n* = 11). Cells were stabilized at a lower ratio than in **a**. Interestingly, when the periodic stimulation was stopped, the cell population split into two groups, each attracted by a stable equilibrium state. This demonstrates that the cell population was close to the frontier between basins of attraction. **g** In silico periodic stabilization of a population of cells (*n* = 16) for the same dynamic stimulation shown in **f**. See Supplementary Fig. 8 for additional experiments

Controlling several cells simultaneously would require in principle the application of specific inputs for each cell to account for cell-cell variability and the stochastic nature of gene expression. This is especially the case in the context of a bistable system for cells states near the separatrix, for which small, stochastic differences between cells can lead to different commitment. Coming back to the inverted pendulum control problem, this would mean that several inverted pendulums differing in mass and length could be stabilized in their upward position by applying the same mechanical forces. Actually, physics tells us that this is possible using dynamic stabilization, the most famous example being the Kapitza method:^19^ a pendulum whose base is subjected to fast vertical oscillations becomes stable in its upward, unstable equilibrium position. More generally, periodic forcing may be used to stabilize dynamically an unstable system. Yet, to our knowledge, dynamic stabilization through periodic forcing of a bistable genetic circuit has never been reported. To explore this idea further, cells were subjected to periodic stimulation of aTc and IPTG. For too fast (Supplementary Fig. 8), or too slow (more than a few hours, Fig. 1) periodic stimulations, we did not observe any stabilization: cells eventually committed to either one of their two possible (stable) fates. However, for carefully chosen periods (Fig. 4a, c, Supplementary Fig. 8, and Supplementary Movies 3 and 4), all cells in the field of view followed large trajectories in a region where both LacI and TetR were expressed at comparable levels. These cells did not commit to either of the two stable states. The cells were indeed in an unstable region, since as soon as the periodic stimulations were interrupted, the cells were attracted to either one of the two stable equilibria (Fig. 4a, c). Comparable results were obtained using the numerical model to simulate cells behaviors (Fig. 4b). Moreover, thanks to the model, we could obtain an idea of the stabilization mechanism at play. The vector field in which cells evolve is periodically transformed (Supplementary Fig. 5), alternating between two situations in which there is only one stable state at a given time, that corresponds to a full aTc or a full IPTG induction. Yet, cells commitment dynamics ( ~ 3–4 h, Fig. 1c) are slower than the periodic change of the state space, and as a result, cells effectively follow the time-averaged vector field (i.e., averaged over one period of stimulation) represented in Fig. 4d. This sheds light on the origin of the synchronized behavior of the cell population (Fig. 4a, f): the time-averaged vector field presents a unique effective equilibrium point, acting like a global attractor for all cells, irrespectively of their phenotypic differences. Accordingly, we found that different ratio of inducer durations drove cells to oscillate in different locations in the state space (Fig. 4e) and, after release of the periodic forcing, to fall in the two basins of attractions in variable proportions (Fig. 4a, f and Supplementary Fig. 9). Taken together, we demonstrated that periodic forcing can be used to force a population of cells to remain near a specific location in the state space despite their phenotypic differences. Such method is less versatile since it is not possible to decide exactly at which positions the cells will be maintained, but it can be readily applied for a population of cells, for example, to reset their states or prevent their commitment to a cell fate (as shown in Fig. 4).

## Discussion

Bistable systems are known to play a central role in cellular decision-making, starting with cellular differentiation. Indeed, decision processes can be understood as the continuous transformation of a stable equilibrium, in which cells reside in an unstable state, separating the state space into several basins of attraction corresponding to the different possible futures of the cells^11,14,20,21^. Therefore, in addition to its importance as a test assay of the control of complex circuits at the single-cell level, the control methods outlined in this article are relevant to externally drive cellular decisions, cell fate and possibly differentiation dynamics.

Of course, the ability to remotely pilot complex gene regulatory dynamics requires to be able to perturb them. Here, this was made possible by using two chemicals which are well known to interact with our implementation of the genetic toggle switch. Actually, many gene regulatory networks can be activated or inhibited by addition of chemicals to the extracellular environment, and an important avenue of research in “cybergenetics” is to find to what extent cells can be controlled using such chemicals despite their potential large spectrum of action on cellular processes. An alternative approach is to use modern gene editing techniques to implement synthetic gene expression inducers within the cells. For example, with optogenetics, gene expression can be directly controlled by sending light to the cells.

Another important finding of our study is the fact that periodic perturbations could reset and maintain the state of a population of cells in an “undecided” state from which several cell fates were possible, once external forcing was released. This result can be explained by a simple mechanism: when subjected to rapid, periodic stimulations, the comparatively slower genetic circuit approximately follows a dynamic process corresponding to a time-averaged phase portrait, which presents a single attracting point. Taken together, our results demonstrate that this simple strategy can be applied to collectively reset the state of a population of cells bearing a bistable circuit, without monitoring the cells position in the state space. Moreover, this periodic forcing stabilization effect also sheds a new light on the role of pulsatile systems^22^ in biology, which have been related to the maintenance of pluripotent states. For example, oscillations in transcription factor levels have been shown to be essential for the maintenance of neural progenitor cells in non-differentiated states^23,24^. Thus, we anticipate that similar dynamic stabilization methods could be generalized to investigate cell maintenance and differentiation.

## Methods

### Plasmids and strains

The *E. coli* strain was based on the K-12 BW25113 background with the *fliA*, *lacY*, *acrA* and *acrB* genes knocked out. Standard P1 phage transductions were used to delete the *lacY* gene. The presence of LacY permease is known to confer hysteretic behavior to the lactose induction system 25. The a*crA* and *acrB* genes were deleted using a modified Wanner chromosomal deletion/integration protocol on the BW25113 parental strain, followed by transfer of the double deletion through P1 phage transduction into the *fliA*, *lacY* strain. This additional double mutation was necessary to prevent cell adaptation to sustained aTc levels. Indeed, the acriflavine efflux pumps encoded by the *acrA* and *acrB* genes are suspected to allow *E. coli* cells to adapt to high concentrations of tetracycline. To create a toggle switch circuit with bistable behavior, we constructed a library based on the original LacI/TetR toggle switch design. Plasmids in the library only differed in their ribosome-binding site sequences (RBS). Plasmid assembly was performed via Modular Cloning^25^ and a low copy number backbone plasmid derived from the ACYC family producing about 10–12 plasmids per cell^26^. This reduced the expression burden on the cells and increased the fidelity of the Modular Cloning method. The *lacI* and *tetR* genes and their promoters were placed in an operon structure with the *mKate2* and *mEGFP* reporter genes, respectively. A plasmid that displayed a robust toggle switch behavior was selected and used for all experiments presented in this article (see Supplementary Fig. 1). This circuit had identical RBS sequences for both operons. The plasmid sequence is available as Supplementary Material (Supplementary Note 1). Strains and plasmids are available upon request.

### Microfluidics

Experiments were performed using a microfluidic device containing an array of parallel chambers connected to a large channel^27^. Nutrients and chemicals of interest are supplied to cells growing in the chambers via diffusion from the large channel. Growth chambers were micro fabricated using electron-beam lithography on SU-8 resin (25 µm long, 1.5 µm wide,  ~ 1 µm high). Larger flow channels were fabricated using standard soft-lithography techniques. Microfluidic chips were molded from the master wafer in polydimethylsiloxane (PDMS). After plasma activation, the PDMS chip was bonded to a glass slide. Cells were transfected with the toggle switch plasmid, grown overnight in LB medium at 37 °C with 50 µg ml^−1^ of chloramphenicol, and loaded into the chambers by centrifugation on a spin-coater using a dedicated 3D printed device. Cells were allowed to recover in the microfluidic chambers for 4 h at 37 °C under a constant flow of LB medium supplemented with 5 g l^−1^ F-127 pluronic to passivate the PDMS surfaces and prevent cell adhesion. Initially, the medium also contained 1 mM IPTG, unless specified otherwise. Thus, cells always started in the GFP-dominant state. Chemical inducers (aTc and IPTG) were added to the growth media as required using a set of three solenoid valves (The Lee Company). The valves were connected to the microfluidic chip, inter-connected, and connected to the growth media, growth media with IPTG (1 mM) and growth media with aTc (100 ng ml^−1^), so that three-way pulse-width-modulation and mixing were possible to deliver the desired concentrations of the two inducer molecules to the *E. coli* cells in the growth chambers. The function of the valve mixing setup was verified by mixing fluorescein and rhodamine solutions and measuring fluorescence levels within the microfluidic chip (Supplementary Fig. 2).

### Microscopy and image analysis

Images were acquired using an Olympus IX71 microscope equipped with an Olympus 100× UPlanSApoPh3 oil objective, CoolSNAP HQ2 CCD Camera, PI piezo *Z*-stage, and Prior H117 motorized XY stage. A HBO 103 W/2 mercury short-arc lamp and mEGFP and mCherry filter cubes were used for fluorescence imaging. μManager^28^ was used for time-lapse acquisition and MATLAB for image analysis, real-time control and actuation of microfluidic valves. A custom image analysis program was written to track cells that exploits the properties of the ‘mother machine’ microfluidic topology. Fluorescence levels were measured within a small rectangular region of interest located at the end of each chamber where a single cell is expected to be trapped at all times. The evolution of several individual cells could be precisely and robustly followed for extended periods of time ( > 15 h). Cells that filamented or did not grow were not considered for control experiments and analysis (pre-established criterion). In total, we run 7 single-cell control and 14 dynamic stimulation experiments. The experimental data and time-lapse microscopy images are available upon request.

### Models and numerical simulations

Models are based on pseudo-reactions that represent transcription, translation, and degradation/dilution due to growth. Depending on the problem, a deterministic or stochastic interpretation of this set of pseudo-reactions was employed. Mathematically, the deterministic interpretation of the above pseudo-reactions results in an ordinary differential equation (ODE) model, integrated with a stiff ODE solver (ode23s in Matlab). The stochastic interpretation results in a continuous time Markov chain model, simulated using the first reaction method version of the Gillespie algorithm^29^. The propensity functions are given by the rate laws of the pseudo-reactions. To obtain a good fit to the calibration data (Supplementary Fig. 3), we had to incorporate to our model relatively slow exchanges of IPTG in and out of the cell. This lead us to a first inducer exchange model: *aTc* = *u*_aTc_ and ${\rm{d}}{IPTG}/{\rm{d}}t = {k_{{\rm{IPTG}}}}\left( {{u_{{\rm{IPTG}}}} - {{IPTG}}} \right)$, where *u*_aTc_ and *u*_IPTG_ denote the concentrations of the external inducers. Our control target was identified by fitting this model to calibration data (Supplementary Figs. 3–5). Model fitting was performed using the global optimization tool CMAES^30^. The objective was to minimize the mean squared relative deviations between model predictions obtained with the deterministic interpretation of the model and the averaged measured fluorescence levels. However, to obtain a model also consistent with control experiments, we had to assume a non-symmetrical exchange of aTc and IPTG in and out of the cell, leading to a more complex inducer exchange model:

$$\begin{array}{l}\\ {\rm{d}}aTc/{\rm{d}}t = \left\{ {\begin{array}{*{20}{c}}{k_{\rm aTc}^{\rm in}\left( {{u_{\rm aTc}} - aTc} \right),{\rm{if}}\,{u_{\rm aTc}}  >aTc} \\ \\ {k_{\rm aTc}^{\rm out}\left( {u_{\rm aTc} - {{aTc}}} \right),{\rm{if}}\,{u_{\rm aTc}} \le aTc} \\ \end{array}} \right.\\ \\ \\ {\rm{d}}{IPTG}/{\rm{d}}t = \left\{ {\begin{array}{*{20}{c}}{k_{\rm IPTG}^{in}\left( {{u_{\rm IPTG}} - {IPTG}} \right),{\rm{if}}\,{u_{{\rm{IPTG}}}}  >{IPTG}} \\ \\ {k_{\rm IPTG}^{\rm out}\left( {u_{\rm IPTG} - {{{IPTG}}}} \right),{\rm{if}}\,{u_{{\rm{IPTG}}}} \le {IPTG}} \\ \end{array}} \right.\\ \end{array}$$

This aspect of the dynamics was not strongly constrained in calibration experiments that display only low frequency inducer changes. Strong binding of effectors to repressors or adsorption effects could lead to such apparent non-symmetrical exchange rates. With the exception of Supplementary Figs. 3–5, all in silico results have been obtained with this reference model (Figs. 1–4 and Supplementary Fig. 7). This extension of the model necessitated the manual adjustment of several parameters, however, the location of equilibrium points in the state space was only marginally changed (compare Fig. 1f with Supplementary Fig. 4). All parameters are provided in Supplementary Table 1.

### Control method

Two independent controllers drive the TetR-GFP level by adjusting the IPTG concentration and the LacI-RFP level by adjusting the aTc concentration. Control errors are defined as the differences between target and measured fluorescence levels. For the PI controllers, the concentration of the inducers was set based on the current deviation from the target and the past deviations to the target, each weighted by two coefficients.

$${u_{\rm aTc}}\left( t \right) = {u_{\rm aTc}^0} + {\rm K}_{\rm P}^{\rm L}\left( {{{Lac}}{{{I}}^{\rm{*}}} - {{LacI}}\left( t \right)} \right) + {\rm K}_{\rm I}^{\rm L}\mathop {\int }\nolimits_{\hskip-2pt 0}^t \left( {{{Lac}}{{{I}}^{\rm{*}}} - {{LacI}}\left( s \right)} \right){\rm{d}}s$$

$${u_{\rm IPTG}}\left( t \right) = {u_{\rm IPTG}^0}+{\rm K}_{\rm P}^{\rm T}\left( {{{Tet}}{{{R}}^{\rm{*}}} - {{TetR}}\left( t \right)} \right) + {\rm K}_{\rm I}^{\rm T}\mathop {\int }\nolimits_{\hskip-2pt 0}^t \left( {{{Tet}}{{{R}}^{\rm{*}}} - {{TetR}}\left( s \right)} \right){\rm{d}}s$$

Additional constraints stipulated that inducer concentrations remain between admissible bounds:

$$u_{\rm aTc}^{{\rm{min}}} = u_{{\rm{IPTG}}}^{{\rm{min}}} = 0$$, $$u_{\rm aTc}^{{\rm{max}}} = 50 \,{\rm{ng}}\,{\rm{m}}{{\rm{L}}^{ - {\rm{1}}}}$$, and $$u_{{\rm{IPTG}}}^{{\rm{max}}} = 0.5\,{\rm{mM}}$$.

Lastly, to reduce overshoot caused by the accumulation of the unavoidable initial deviations in the integral term, we neglected the first 2 h in the integral. More precisely, for the integral term, we used for the two repressors, LacI and TetR, either ${\rm K_I^R}\mathop {\int }\nolimits_{{t_d}}^t \left( {{\rm{Rep}}{{\rm{r}}^*} - {\rm{Repr}}\left( s \right)} \right){\rm{d}}s$ with *t*_*d*_ = 2 h, if *t*>*t*_*d*_, or 0, if $t \le {t_d}$. The bang-bang controller is extremely simple. The maximal or minimal inducer concentration is applied based on the sign of the current deviation to the target.

$${u_{\rm aTc}}\left( t \right) = \left\{ {\begin{array}{*{20}{c}}\\ {u_{\rm aTc}^{{\rm{max}}},{\rm{if}}\,{{Lac}}{{{I}}^*}  >{{LacI}}\left( t \right)} \\ \\ {u_{\rm aTc}^{{\rm{min}}},{\rm{if}}\,{{Lac}}{{{I}}^*} \le {{LacI}}\left( t \right)} \\ \end{array}} \right.\,{\rm{and}}\,{u_{{\rm{IPTG}}}}\left( t \right) = \left\{ {\begin{array}{*{20}{c}}\\ {u_{\rm IPTG}^{{\rm{max}}},{\rm{if}}\,{{Tet}}{{{R}}^*}  >{{TetR}}\left( t \right)} \\ \\ {u_{{\rm{IPTG}}}^{{\rm{min}}},{\rm{if}}\,{{Tet}}{{{R}}^*} \le {{TetR}}\left( t \right)} \\ \end{array}} \right.$$

again using $u_{\rm aTc}^{{\rm{min}}} = u_{{\rm{IPTG}}}^{{\rm{min}}} = 0$, $u_{\rm aTc}^{{\rm{max}}} = 50\,{\rm{ng}}\,{\rm{m}}{{\rm{L}}^{ - {\rm{1}}}}$, and $u_{{\rm{IPTG}}}^{{\rm{max}}} = 0.5\,{\rm{mM}}$.

### In silico control experiments

A stochastic interpretation of the model was used to test the capacity of control and periodic stimulation strategies to drive a cell. In the case of closed-loop control, we simulated the (stochastic) behavior of a cell subjected to the control actions made by the PI or bang-bang controllers defined above. To investigate the behavior of the other cells in the population, we created in silico a population of cells, each behaving stochastically and independently, and predicted their behaviors when subjected to the environment of the controlled cell. In the case of periodic stimulation, we simply simulated the behavior of a population of cells, each behaving stochastically and independently, when subjected to alternating environments. Matlab code for the models and the control experiments are available upon request.

### Data availability

The data sets generated and/or analyzed for this study (DOI 10.5281/zenodo.894275) are available on our GitHub repository (https://github.com/Lab513/CyberSwitch) and directly from the corresponding authors on reasonable request.

## Electronic supplementary material

Supplementary Information

Peer Review File

Description of Additional Supplementary Files

Supplementary Movie 1

Supplementary Movie 2

Supplementary Movie 3

Supplementary Movie 4
