# Supplementary material for: Balancing a genetic toggle switch by real-time feedback control and periodic forcing
Source: Nat Commun. 2017 Nov 17;8:1671. doi: 10.1038/s41467-017-01498-0 (PMC5693866; doi:10.1038/s41467-017-01498-0)
Supplement: Supplementary file 1 — Supplementary Information [file 41467_2017_1498_MOESM1_ESM.pdf]

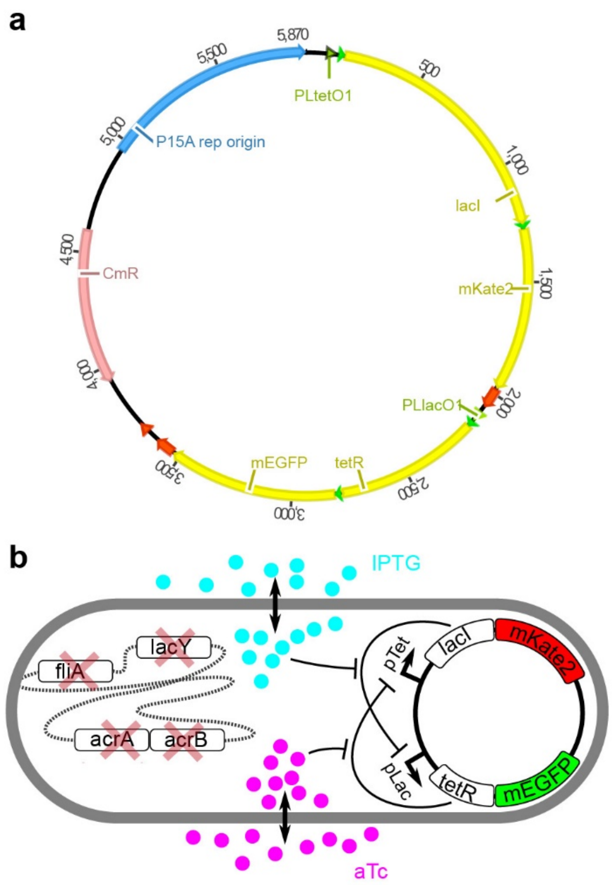

Supplementary Figure 1. Toggle switch cells. a. Map of the toggle switch plasmid. Yellow arrows are coding sequences (*lacI*-*mKate2* and *tetR*-*mEGFP*). Light green arrows are promoters (pLac and pTet). Dark green arrows represent ribosome binding sites (RBS). Red arrows represent terminators. The chloramphenicol resistance cassette and plasmid origin of replication are shown in pink and blue, respectively. **b**. The cellular chassis is based on the K-12 BW25113 background with the *fliA*, *lacY*, *acrA* and *acrB* genes knocked out. IPTG and aTc enter the cell by passive diffusion through the cellular membrane and interact with the LacI and TetR proteins. The cellular state is observed by monitoring the fluorescence levels of mEGPF (TetR) and mKate2 (LacI).

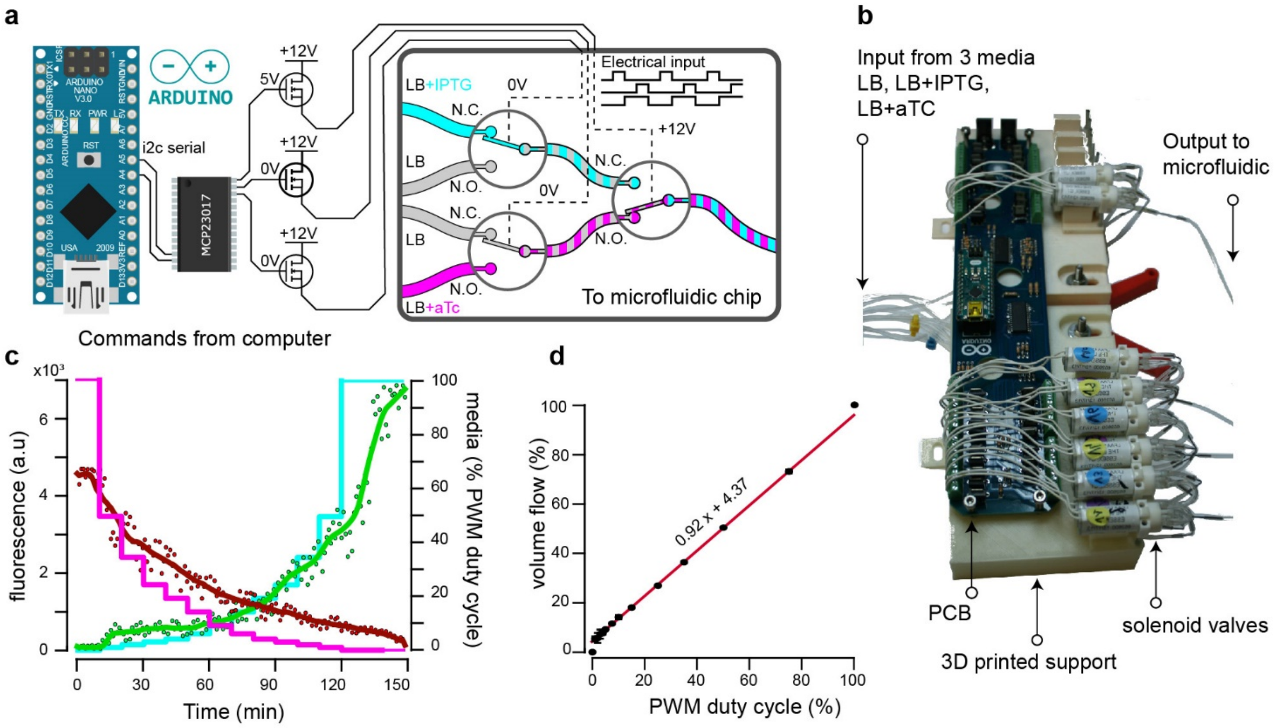

Supplementary Figure 2. Characterization of mixing device. **a**. Sketch of the electronic circuit controlling the fluidic valve and the principle of mixing in Pulse Width Modulation (PWM) mode. **b**. Actual mixing device showing the two triplets of valves required to run two experiments in parallel. **c**. Various concentrations of fluorescein and rhodamine were flowed through the main microfluidic device and the levels of fluorescence in the chambers were monitored. The fluorescence levels closely follow the mixing levels imposed by the valve system. **d**. Calibration experiment: PWM mixing was run over extended periods of time at different duty cycles, and volume consumption was measured.

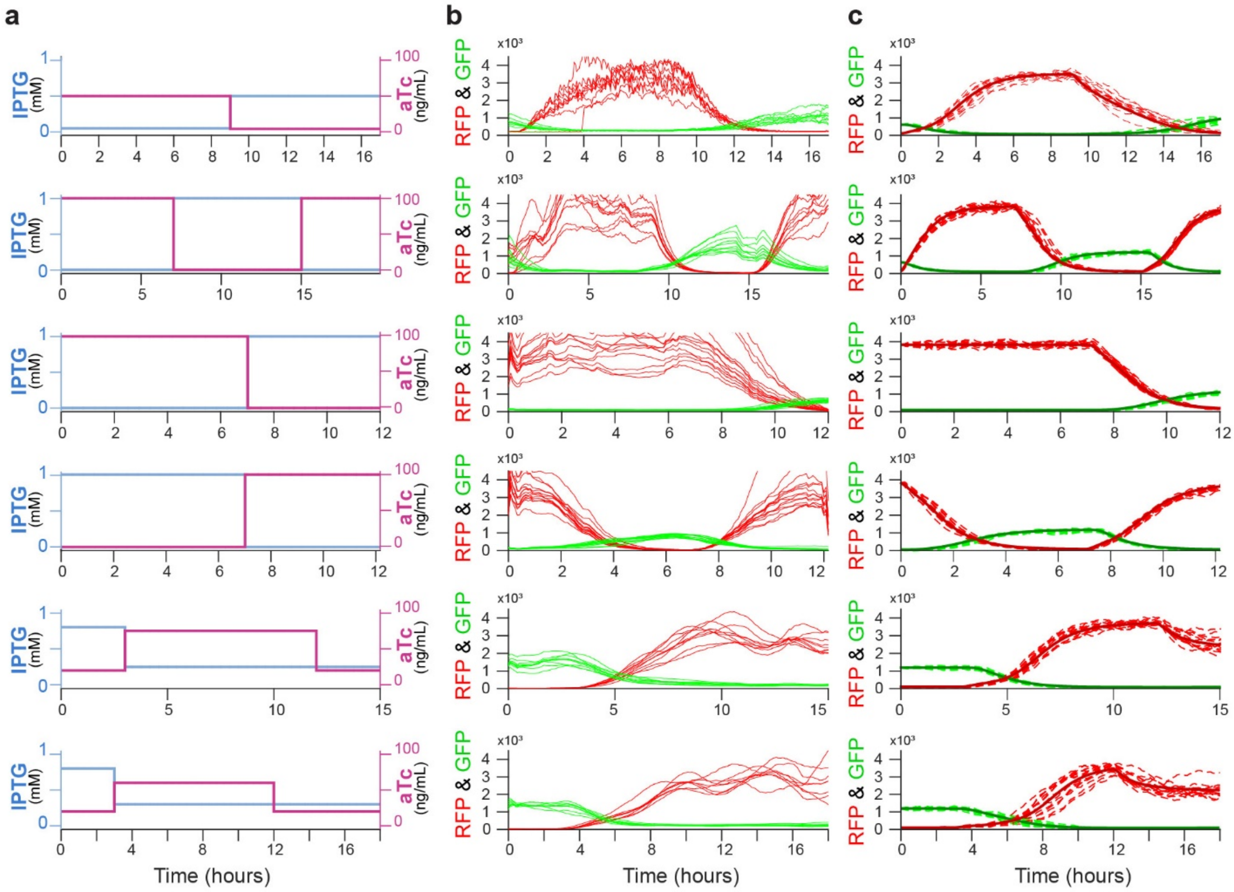

Supplementary Figure 3. Model calibration. a. Several temporal profiles using different levels of aTc and IPTG inducers were used to construct an experimental data set to characterize the behavior of the system. **b**. Corresponding temporal evolution of single cells (n ~ 10 cells per experiments). Red and green lines correspond to single-cell LacI-RFP and TetR-GFP fluorescence levels in arbitrary units. **c.** Predictions obtained using the model fitted to the average behavior of all experiments. Thick red and green curves correspond to the behavior of the deterministic model. Thinner dotted lines correspond to predictions obtained using the stochastic implementation of the model. Naturally, as the stochastic model only captures intrinsic variability and as all simulations started from the same initial state, we expected that the variability of the simulated cells was smaller than the one of the observed cells.

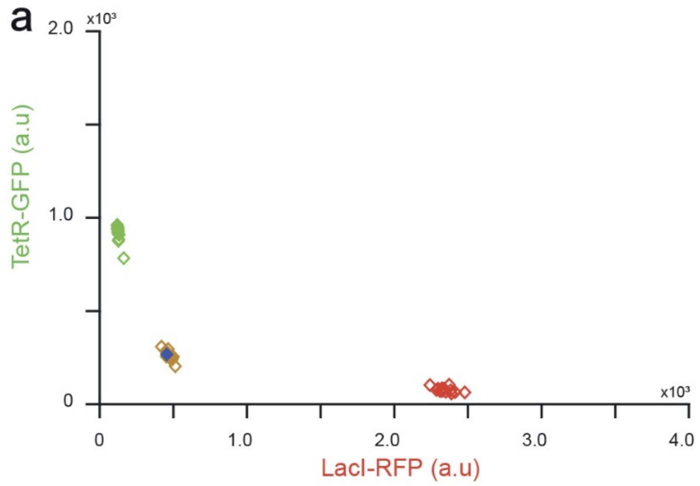

Supplementary Figure 4. Capacity of the characterization data to constrain the locations of the equilibrium points of the deterministic model. Distribution of the equilibrium points in the protein space under reference conditions as predicted using the 15 best-fitting parameter sets when the optimization was iterated 30 times. LacI-dominant and TetR-dominant stable equilibria are represented by red and green diamonds, whereas unstable equilibria are represented by yellow diamonds. The set-point used in control experiments is represented by a blue square.

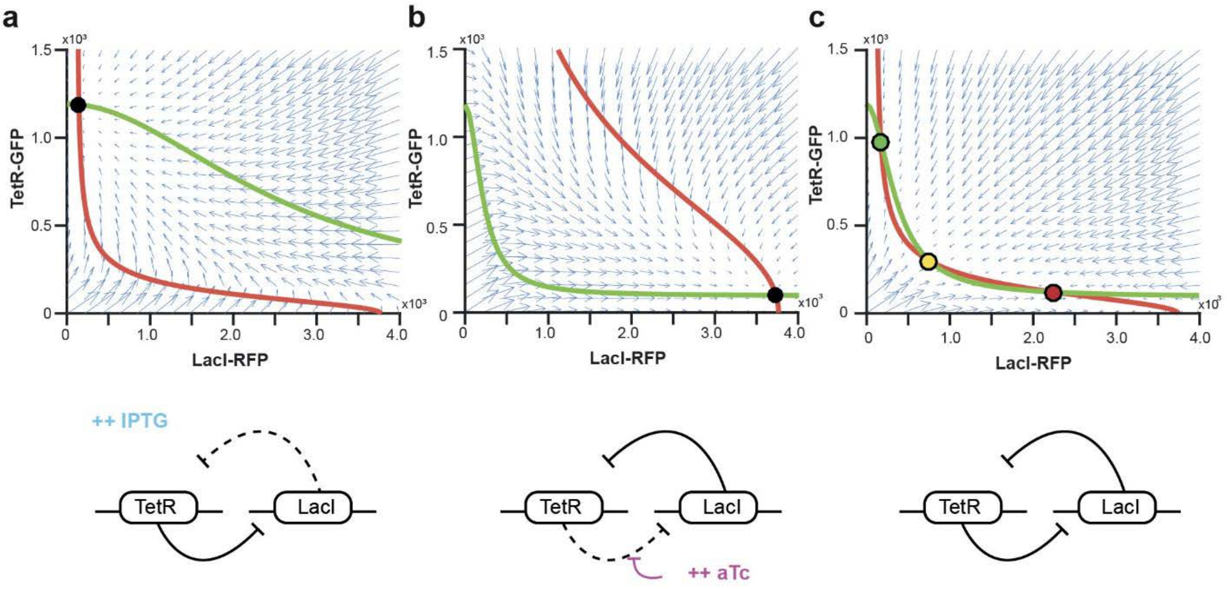

Supplementary Figure 5. Influence of IPTG and aTc on the bistable behavior of the toggle switch. a. State space in the presence of an excess of IPTG (1 mM) as predicted by the numerical model. Addition of IPTG turns off the repression of the *tetR* gene by LacI. As a result, TetR is fully expressed and the system only presents one stable, attractive state: all cells will turn green eventually. **b.** Similarly, an excess of aTc (100 ng.mL^{-1}) turns off the repression of the *lacI* gene by TetR. As a result, LacI is fully expressed and cells will eventually reach the unique stable state (high level of LacI-RFP). **c.** An interesting case occurs when both branches are in competition (aTc = 20 ng.mL^{-1}, IPTG = 0.25 mM), so that there are two stable equilibria and one unstable equilibrium (see Figure 1 and Main text) and reproduced here numerically using a deterministic model. The red and green curves shown in (**a-c)** are the LacI and TetR nullclines computed using the fitted model under different operating conditions. By definition, equilibria are located at nullcline intersections.

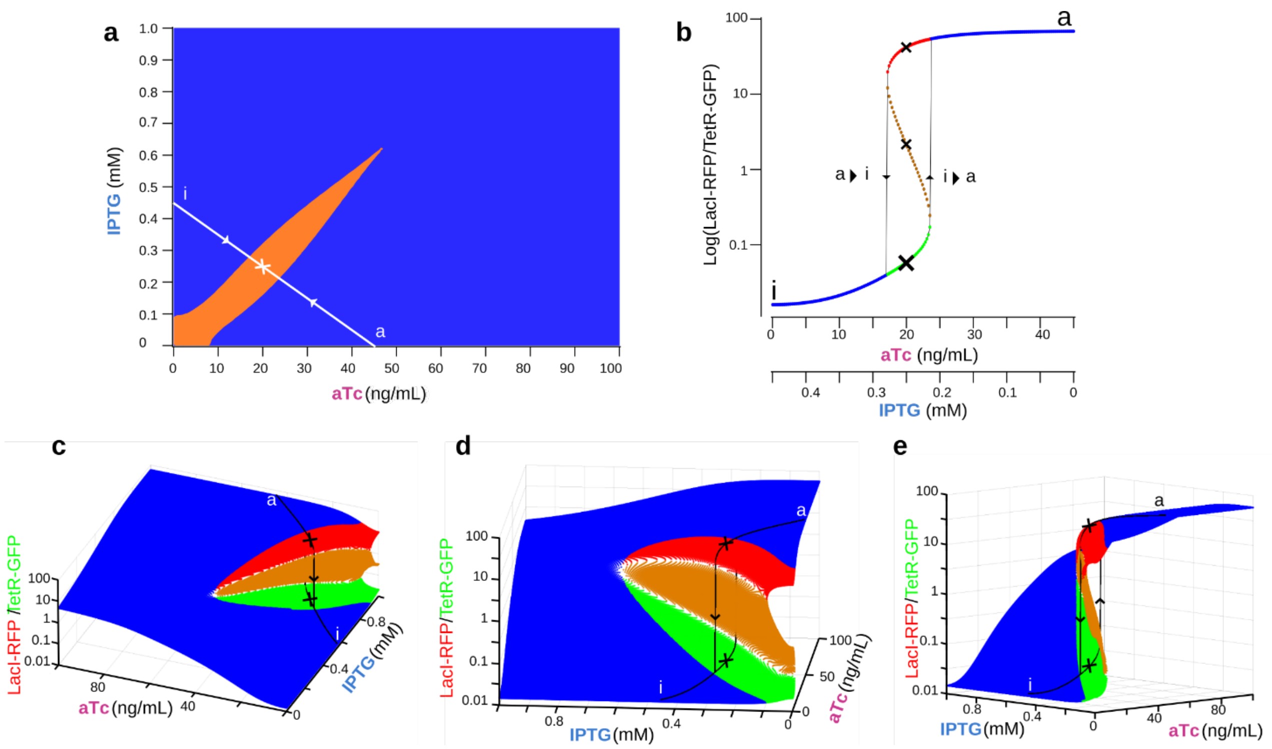

Supplementary Figure 6. Stability analysis of the toggle switch circuit in different environments. a. Representation of the stability of the circuit as a function of inducer concentrations. Blue and orange regions represent the conditions under which the system is mono- or bi-stable, respectively. The white segment going from point a (full aTc; aTc = 45 ng.mL^{-1}, IPTG = 0) to point i (full IPTG; aTc = 0, IPTG = 0.45 mM) passes through the reference concentrations for this study (white cross, aTc = 20 ng.mL^{-1} and IPTG = 0.25 mM). **b**. Logarithm of the LacI-RFP/TetR-GFP ratio of the stable and unstable equilibrium points are represented for different concentrations of aTc and IPTG along the white segment of panel **(a)**. Monostable points are represented in blue. LacI-dominant and TetR-dominant stable points of the bistable region are represented in red and green, respectively. Unstable equilibrium points of the bistable region are represented in orange. Along this line, the system shows hysteresis. **c-e**. Same representation of the stability of the equilibria of system as in **(b)** represented in the aTc/IPTG plane. Different views of the cusp-catastrophe curve. The hysteresis line from panel **(a)** and **(b)** is represented in black.

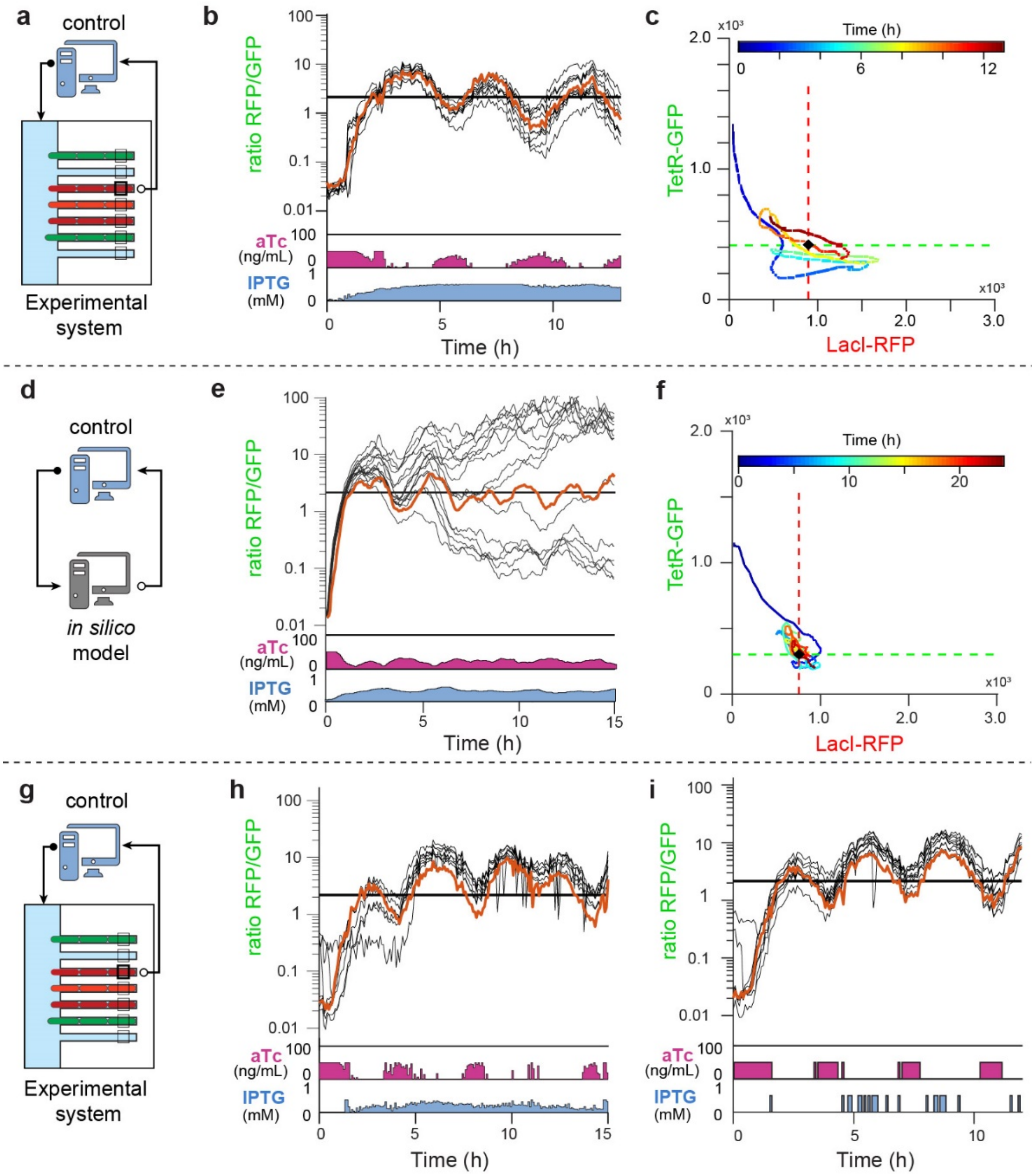

Supplementary Figure 7. Other example of PI control experiments. a. Experimental control runs. **b.** Ratio of red (LacI-RFP) and green (TetR-GFP) fluorescence levels as a function of time using a double PI controller to drive protein expressions. The parameters of the PI dual controller are $K_P^L = 5.0\ 10^{-2}$, $K_I^L = 2.0\ 10^{-4}\ s^{-1}$, $K_P^T = 2.5\ 10^{-2}$, $K_I^T = 6.94\ 10^{-4}\ s^{-1}$ (see Methods). Thick orange and thin black lines represent controlled and non-controlled cells (n = 10), respectively. **c**. Trajectory of the controlled cells in the state space. The cell starts from a high TetR-GFP state and then moves towards the target point (black diamond) while describing large oscillations around the target. Time is color coded from t = 0 h (blue) to t = 12 h (red). **d.** *In silico* control runs. **e**. Ratio of red (LacI-RFP) and green (TetR-GFP) fluorescence levels as a function of time using a double PI controller to drive simulated cells (n = 20). The PI parameters are $K_P^L = 5.0\ 10^{-2}$, $K_I^L = 2.0\ 10^{-4}\ s^{-1}$, $K_P^T = 2.5\ 10^{-2}$, $K_I^T = 6.94\ 10^{-4}\ s^{-1}$. **f**. Trajectory of the controlled cell for *in silico* control experiments. **g.** Experimental control runs. **h.** Ratio of red (LacI-RFP) and green (TetR-GFP) fluorescence levels of single cells (n = 8) as a function of time using a double PI controller to drive protein expressions. The PI parameters are $K_P^L = 5.0\ 10^{-2}$, $K_I^L = 2.0\ 10^{-4}\ s^{-1}$, $K_P^T = 2.5\ 10^{-2}$, $K_I^T = 6.94\ 10^{-4}\ s^{-1}$. **i.** Ratio of red (LacI-RFP) and green (TetR-GFP) fluorescence levels of single cells (n = 8) as a function of time using a double Bang-bang controller to drive protein expressions.

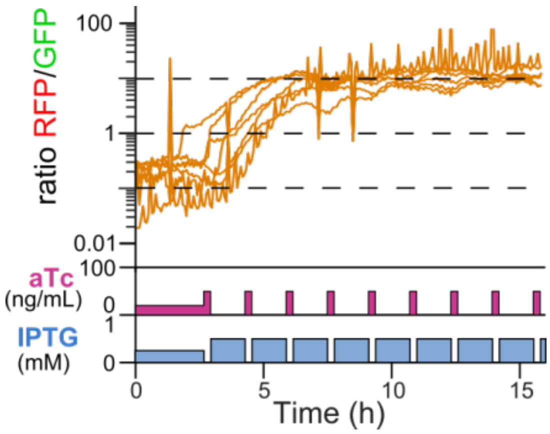

Supplementary Figure 8. Too Fast periodic forcing experiments. Ratio of the red (LacI-RFP) and green (TetR-GFP) fluorescence levels of cells subjected to periodic stimulations with alternating high aTc and high IPTG concentrations. The cells were grown in 0.25 mM IPTG and 20 ng.mL^{-1} aTc prior to the experiment and during the first 2.5 hours. They were then subjected to alternations of 0.5 mM IPTG for 75 min, 50 ng.mL^{-1} aTc for 15 min. The cells do not remain in a state of balanced expression, and instead drift to the LacI-RFP high state.

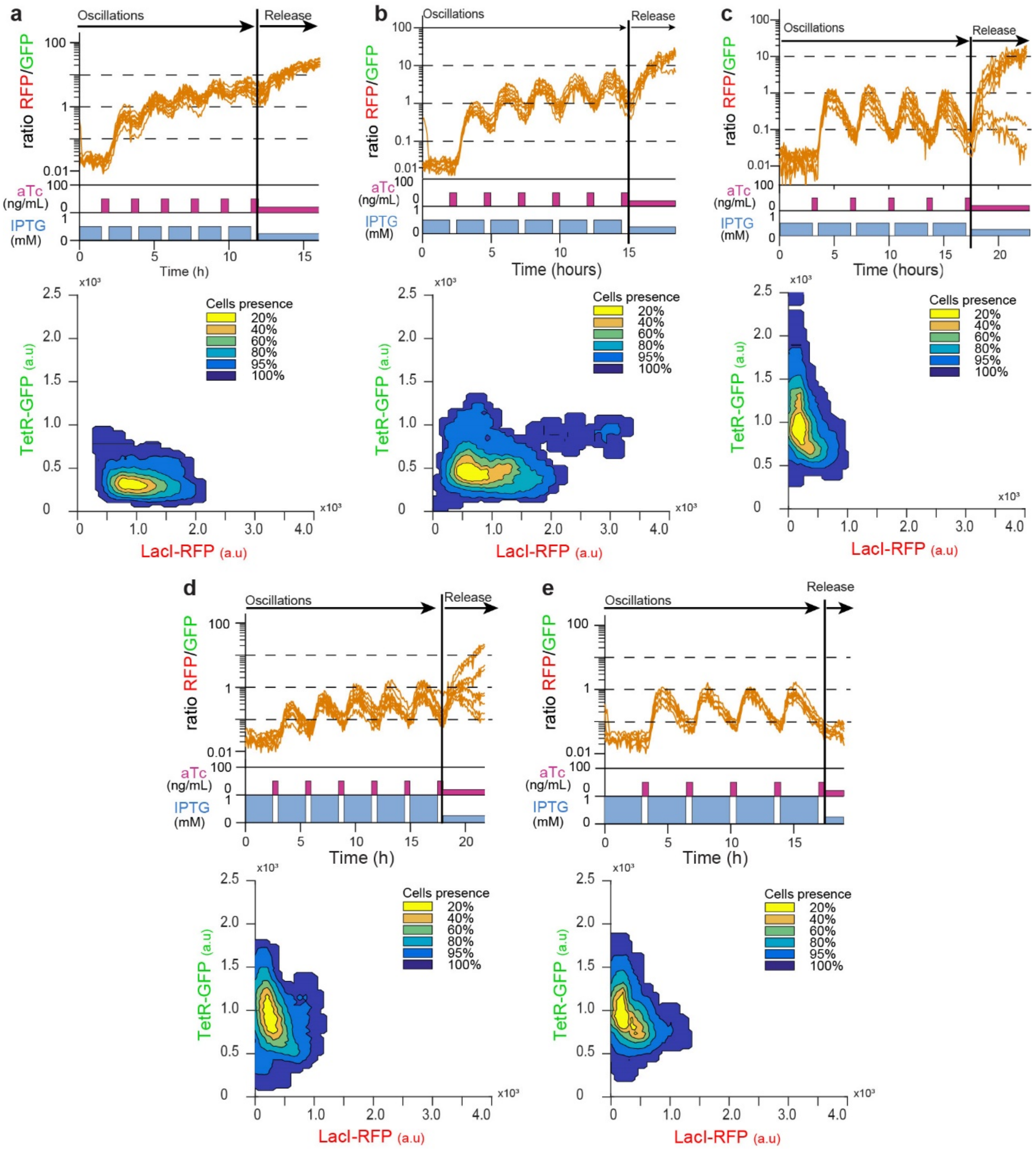

Supplementary Figure 9. Additional periodic forcing experiments. Ratio of the red (LacI-RFP) and green (TetR-GFP) fluorescence levels of cells subjected to periodic stimulations with alternating high aTc and high IPTG concentrations. All cells present a similar behavior and remain in a state of balanced expression. Cell probability of presence for the two last input periods is shown below. Experimental conditions were: **(a)** 0.5 mM IPTG for 90 min, 50 ng.mL^{-1} aTc for 30 min, n=12; **(b)** 0.5 mM IPTG for 120 min, 50 ng.mL^{-1} aTc for 30 min, n = 10; **(c)** 0.5 mM IPTG for 180 min, 50 ng.mL^{-1} aTc for 30 min, n = 10; **(d)** 1.0 mM IPTG for 150 min, 50 ng.mL^{-1} aTc for 30 min, n = 9; **(e)** 1.0 mM IPTG for 180 min, 50 ng.mL^{-1} aTc for 30 min, n = 7. As expected, the average ratio shifted to RFP when the periodic forcing favored aTc over IPTG. At some point, periodic forcing was stopped to observe release of the cells. Cells were **(a-b)** subsequently attracted to the RFP dominant state; **(c-d)** split into two sub-populations attracted to either RFP or GFP dominant states; or **(e)** attracted to the GFP dominant state.

Supplementary Table 1

Transcription rates (mRNA min^{-1})	κ_L^{m0}	3.20e-2 (3.045e-1)	*plac* regulation by TetR	θ_{LacI}	31.94 (124.9)
	κ_T^{m0}	1.19e-1 (3.313e-1)		η_{LacI}	2.00 (2.00)
	κ_L^{m}	8.30 (13.01)		θ_{IPTG}	9.06e-2 (2.926e-1)
	κ_T^{m}	2.06 (5.055)		η_{IPTG}	2.00 (2.00)
Translation rates (a.u. $mRNA^{-1}$ min^{-1})	κ_L^{p}	9.726e-1 (6.606e-1)	*ptet* regulation by LacI	θ_{tetR}	30.00 (76.40)
	κ_T^{p}	1.170 (5.098e-1)		η_{TetR}	2.00 (2.152)
Degradation rates (min^{-1})	g_L^{m}	1.386e-1		θ_{aTc}	11.65 (35.98)
	g_T^{m}	1.386e-1		η_{aTc}	2.00 (2.00)
	g_L^{p}	1.65e-2	IPTG exchange rate (min^{-1})	$\kappa_{IPTG}^{\text{in}}$	2.75e-2 (4.00e-2)
	g_T^{p}	1.65e-2		$\kappa_{IPTG}^{\text{out}}$	1.11e-1 (4.00e-2)
			aTc exchange rate (min^{-1})	κ_{aTc}^{in}	1.62e-1 (N/A)
				$\kappa_{aTc}^{\text{out}}$	2.00e-2 (N/A)

Supplementary Table 1. Parameters for the toggle switch model. Threshold parameters are expressed in arbitrary fluorescence units (a.u.) for proteins (θ_{LacI} and θ_{TetR}), ng.mL^{-1} for aTc (θ_{aTc}), and mM for IPTG (θ_{IPTG}). The values of 4 parameters were fixed. mRNA degradation rates were fixed based on typical half-life of 5 minutes for mRNAs in *E. coli* (1). Protein apparent degradation rates were set based on estimates of cell cycle times in our experiments (~42 min). We therefore assumed that dilution due to growth dominates over actual protein degradation. Note that the significant difference between the mRNA and protein apparent degradation rates allowed us to employ a quasi-steady state assumption in which mRNAs are assumed to be at the steady state levels set by the concentration of the repressors. The dynamics of the 4D system could then be represented in the 2D protein space. For the sake of simplicity, in the main text the latter is called the state space. Realistic initial values for the search were obtained as explained below. Previous work has quantified that the number of mRNAs produced by a fully induced *lac* promoter is ~60 per cell (2). Then the comparison of regulatory ranges and leakage levels given in (3) led to us estimate that the transcription rate of a fully induced *tet* promoter is ~25% higher than the one of *plac*. This provided us with estimates of transcription rates. Lastly, an estimate of translation rates in arbitrary fluorescence units can be deduced from fluorescence levels at maximal induction (3700 a.u. for LacI and 1200 a.u. for TetR) and previous parameter estimates. Global optimization was then used to fit model predictions to calibration data, leading to a first model (parameter values in parenthesis). The addition of non-identical exchange rates in and out of the cell called for a recalibration of the parameters. This has been done by global optimization followed by limited manual adjustments, and has led to the reference model. Lastly, initial conditions were the steady state concentrations that correspond to the pre-incubation media (1 mM IPTG and no aTc, unless specified otherwise).

Supplementary Note 1

```
LOCUS       Toggle_switch      7405 bp    DNA     circular UNA 15-SEP-2017
DEFINITION  Concatenation of 2 sequences.
ACCESSION   urn.local...h-829ke6r
VERSION     urn.local...h-829ke6r
KEYWORDS    .
SOURCE
  ORGANISM
            .
FEATURES             Location/Qualifiers
     misc_feature    21..70
                     /note="Geneious type: Homology"
     protein_bind    111..129
     protein_bind    130..135
     protein_bind    136..153
     protein_bind    153..158
     RBS             169..185
     CDS             186..878
     misc_feature    879..896
                     /note="Geneious type: linker"
     CDS             897..2018
     terminator      2023..2111
     protein_bind    2138..2154
     protein_bind    2155..2160
     protein_bind    2161..2177
     protein_bind    2178..2183
     RBS             2195..2211
     CDS             2212..2925
     misc_feature    2926..2943
                     /note="Geneious type: linker"
     CDS             2944..3597
     terminator      3602..3683
     misc_feature    complement(3729..3776)
                     /note="natural FRT site"
     CDS             complement(3789..4583)
                     /note="kanamycin resistance"
                     /codon_start=1
                     /transl_table=11
                     /product="Tn5 neomycin phosphotransferase"
                     /protein_id="AAL02037.1"
                     /db_xref="GI:15554336"

/translation="MIEQDGLHAGSPAAWVERLFGYDWAQQTIGCSDAAVFRLSAQGR

PVLFVKTDLSGALNELQDEAARLSWLATTGVPCAAVLDVVTEAGRDWLLLGEVPGQDL

LSSHLAPAEKVSIMADAMRRLHTLDPATCPFDHQAKHRIERARTRMEAGLVDQDDLDE

EHQGLAPAELFARLKARMPDGEDLVVTHGDACLPNIMVENGRFSGFIDCGRLGVADRY
                     QDIALATRDIAEELGGEWADRFLVLYGIAAPDSQRIAFYRLLDEFF"
     misc_feature    complement(4951..4985)
                     /note="distal 35-nt of natural FRT site"
     misc_feature    5039..5088
                     /note="Geneious type: Homology"
     misc_feature    5093..5116
     terminator      5140..5187
     CDS             complement(5407..6066)
     rep_origin      6428..7340
ORIGIN
        1 tgccgaattc ggatccggag atgctcaccg ttaagcagat tgaagcagca aagccgaaag
       61 aaaaaccata cgctgcaaca aatgcctaca actagaattc gagctcggag tccctatcag
      121 tgatagagat tgacatccct atcagtgata gagatactga gcactacttc acacaggact
```

```
 181 actaaatgtc tgagctgatt aaggagaaca tgcacatgaa gctgtacatg gagggcaccg
 241 tgaacaacca ccacttcaag tgcacatccg agggcgaagg caagccctac gagggcaccc
 301 agaccatgag aatcaaggcc gtcgagggcg gccctctccc cttcgccttc gacatcctgg
 361 ctaccagctt catgtacggc agcaaaacct tcatcaacca cacccagggc atccccgact
 421 tctttaagca gtccttccct gagggcttca catgggagag agtcaccaca tacgaagatg
 481 ggggcgtgct gaccgctacc caggacacca gcctccagga cggctgcctc atctacaacg
 541 tcaagatcag aggggtgaac ttcccatcca acggccctgt gatgcagaag aaaacactcg
 601 gctgggaggc ctccaccgag acgctgtacc ccgctgacgg cggcctggaa ggcagagccg
 661 acatggccct gaagctcgtg ggcgggggcc acctgatctg caacttaaag accacataca
 721 gatccaagaa acccgctaag aacctcaaga tgcccggcgt ctactatgtg gacagacgac
 781 tggaaagaat caaggaggcc gacaaagaaa cctacgtcga gcagcacgag gtggctgtgg
 841 ccagatactg cgacctccct agcaaactgg ggcacagatc tggatccgga tcttccgtga
 901 atgtgaaacc agtaacgtta tacgatgtcg cagagtatgc cggtgtctct tatcagaccg
 961 tttcccgcgt ggtgaaccag gccagccacg tttctgcgaa aacgcgggaa aaagtggaag
1021 cggcgatggc ggagctgaat tacattccca accgcgtggc acaacaactg gcgggcaaac
1081 agtcgttgct gattggcgtt gccacctcca gtctggccct gcacgcgccg tcgcaaattg
1141 tcgcggcgat taaatctcgc gccgatcaac tgggtgccag cgtggtggtg tcgatggtag
1201 aacgaagcgg cgtcgaagcc tgtaaagcgg cggtgcacaa tcttctcgcg caacgcgtca
1261 gtgggctgat cattaactat ccgctggatg accaggatgc cattgctgtg gaagctgcct
1321 gcactaatgt tccggcgtta tttcttgatg tctctgacca gacacccatc aacagtatta
1381 ttttctccca tgaagatggt acgcgactgg gcgtggagca tctggtcgca ttgggtcacc
1441 agcaaatcgc gctgttagcg ggcccattaa gttctgtctc ggcgcgtctg cgtctggctg
1501 gctggcataa atatctcact cgcaatcaaa ttcagccgat agcggaacgg gaaggcgact
1561 ggagtgccat gtccggtttt caacaaacca tgcaaatgct gaatgagggc atcgttccca
1621 ctgcgatgct ggttgccaac gatcagatgg cgctgggcgc aatgcgcgcc attaccgagt
1681 ccgggctgcg cgttggtgcg gatatctcgg tagtgggata cgacgatacc gaagatagct
1741 catgttatat cccgccgtta accaccatca aacaggattt tcgcctgctg gggcaaacca
1801 gcgtggaccg cttgctgcaa ctctctcagg gccaggcggt gaagggcaat cagctgttgc
1861 ccgtctcact ggtgaaaaga aaaaccaccc tggcgcccaa tacgcaaacc gcctctcccc
1921 gcgcgttggc cgattcatta atgcagctgg cacgacaggt ttcccgactg gaaagcgggc
1981 aggctgcaaa cgacgaaaac tacgctttag tagcttaagc tttagagatc aagccttaac
2041 gaactaagac ccccgcaccg aaaggtccgg gggttttttt tgaccttaaa aacataaccg
2101 aggagcagac acgctttacg aattcccatg ggagaattg tgagcggata acaattgaca
2161 ttgtgagcgg ataacaagat actgagcaca tacttcacac aggactacta aatgagcaag
2221 ggcgaggagc tgttcaccgg ggtggtgccc atcctggtcg agctggacgg cgacgtaaac
2281 ggccacaagt tcagcgtgtc cggcgagggc gagggcgatg ccacctacgg caagctgacc
2341 ctgaagttca tctgcaccac cggcaagctg cccgtgccct ggcccaccct cgtgaccacc
2401 ctgacctacg gcgtgcagtg cttcagccgc taccccgacc acatgaagca gcacgacttc
2461 ttcaagtccg ccatgcccga aggctacgtc caggagcgca ccatcttctt caaggacgac
2521 ggcaactaca agacccgcgc cgaggtgaag ttcgagggcg acaccctggt gaaccgcatc
2581 gagctgaagg gcatcgactt caaggaggac ggcaacatcc tggggcacaa gctggagtac
2641 aactacaaca gccacaacgt ctatatcatg gccgacaagc agaagaacgg catcaaggtg
2701 aacttcaaga tccgccacaa catcgaggac ggcagcgtgc agctcgccga ccactaccag
2761 cagaacaccc ccatcggcga cggccccgtg ctgctgcccg acaaccacta cctgagcacc
2821 cagtccaagc tgagcaaaga ccccaacgag aagcgcgatc acatggtcct gctggagttc
2881 gtgaccgccg ccgggatcac tctcggcatg gacgagctgt acaagtctgg atccggatct
2941 tcctccagat tagataaaag taaagtgatt aacagcgcat tagagctgct taatgaggtc
3001 ggaatcgaag gtttaacaac ccgtaaactc gcccagaagc taggtgtaga gcagcctaca
3061 ttgtattggc atgtaaaaaa taagcgggct ttgctcgacg ccttagccat tgagatgtta
3121 gataggcacc atactcactt ttgcccttta gaaggggaaa gctggcaaga tttttacgt
3181 aataacgcta aaagttttag atgtgcttta ctaagtcatc gcgatggagc aaaagtacat
3241 ttaggtacac ggcctacaga aaaacagtat gaaactctcg aaaatcaatt agcctttta
3301 tgccaacaag gtttttcact agagaatgca ttatatgcac tcagcgctgt ggggcatttt
3361 actttaggtt gcgtattgga agatcaagag catcaagtcg ctaaagaaga aagggaaaca
3421 cctactactg atagtatgcc gccattatta cgacaagcta tcgaattatt tgatcaccaa
3481 ggtgcagagc cagccttctt attcggcctt gaattgatca tatgcggatt agaaaaacaa
3541 cttaaatgtg aaagtgggtc cgctgcaaac gacgaaaact acgctttagt agcttaagct
3601 ttcggtcagt ttcacctgat ttacgtaaaa acccgcttcg gcgggttttt gcttttggag
3661 gggcagaaag atgaatgact gtccgctcag agaattccca tggggagtgt gtaggctgga
3721 gctgcttcga agttcctata ctttctagag aataggaact tcggaatagg aacttcaaga
3781 tccccttatt agaagaactc gtcaagaagg cgatagaagg cgatgcgctg cgaatcggga
3841 gcggcgatac cgtaaagcac gaggaagcgg tcagcccatt cgccgccaag ctcttcagca
3901 atatcacggg tagccaacgc tatgtcctga tagcggtccg ccacacccag ccggccacag
3961 tcgatgaatc cagaaaagcg gccattttcc accatgatat tcggcaagca ggcatcgcca
```

```
4021 tgggtcacga cgagatcctc gccgtcgggc atgcgcgcct tgagcctggc gaacagttcg
4081 gctggcgcga gcccctgatg ctcttcgtcc agatcatcct gatcgacaag accggcttcc
4141 atccgagtac gtgctcgctc gatgcgatgt ttcgcttggt ggtcgaatgg gcaggtagcc
4201 ggatcaagcg tatgcagccg ccgcattgca tcagccatga tggatacttt ctcggcagga
4261 gcaaggtgag atgacaggag atcctgcccc ggcacttcgc ccaatagcag ccagtccctt
4321 cccgcttcag tgacaacgtc gagcacagct gcgcaaggaa cgcccgtcgt ggccagccac
4381 gatagccgcg ctgcctcgtc ctgcagttca ttcagggcac cggacaggtc ggtcttgaca
4441 aaaagaaccg ggcgcccctg cgctgacagc cggaacacgg cggcatcaga gcagccgatt
4501 gtctgttgtg cccagtcata gccgaatagc ctctccaccc aagcggccgg agaacctgcg
4561 tgcaatccat cttgttcaat catgcgaaac gatcctcatc ctgtctcttg atcagatctt
4621 gatcccctgc gccatcagat ccttggcggc aagaaagcca tccagtttac tttgcagggc
4681 ttcccaacct taccagaggg cgccccagct ggcaattccg gttcgcttgc tgtccataaa
4741 accgcccagt ctagctatcg ccatgtaagc ccactgcaag ctacctgctt tctctttgcg
4801 cttgcgtttt cccttgtcca gatagcccag tagctgacat tcatccgggg tcagcaccgt
4861 ttctgcggac tggctttcta cgtgttccgc ttcctttagc agcccttgcg ccctgagtgc
4921 ttgcggcagc gtgagcttca aaagcgctct gaagttccta tactttctag agaataggaa
4981 cttcgaactg caggtcgacg gatccccgga attaattcgc ttgtggaatt cctcgaggga
5041 gaaatgatgc aatggtgggc ggactggctt gatgagaagg tggagtgacg ctgagcgagg
5101 atgcacatgt gaccgaggga tgccaccgct gagcaataac tagcataacc ccttggggcc
5161 tctaaacggg tcttgagggg tttttttgctg aaacctcagg catttgagaa gcacacggtc
5221 acactgcttc cggtagtcaa taaaccggta aaccagcaat agacataagc ggctatttaa
5281 cgaccctgcc ctgaaccgac gaccgggtcg aatttgcttt cgaatttctg ccattcatcc
5341 gcttattatc acttattcag gcgtagcacc aggcgtttaa gggcaccaat aactgcctta
5401 aaaaaattac gccccgccct gccactcatc gcagtactgt tgtaattcat taagcattct
5461 gccgacatgg aagccatcac agacggcatg atgaacctga atcgccagcg gcatcagcac
5521 cttgtcgcct tgcgtataat atttgcccat agtgaaaacg ggggcgaaga agttgtccat
5581 attggccacg tttaaatcaa aactggtgaa actcacccag ggattggctg agacgaaaaa
5641 catattctca ataaaccctt tagggaaata ggccaggttt tcaccgtaac acgccacatc
5701 ttgcgaatat atgtgtagaa actgccggaa atcgtcgtgg tattcactcc agagcgatga
5761 aaacgtttca gtttgctcat ggaaaacggt gtaacaaggg tgaacactat cccatatcac
5821 cagctcaccg tctttcattg ccatacggaa ctccggatga gcattcatca ggcgggcaag
5881 aatgtgaata aaggccggat aaaacttgtg cttatttttc tttacggtct ttaaaaaggc
5941 cgtaatatcc agctgaacgg tctggttata ggtacattga gcaactgact gaaatgcctc
6001 aaaatgttct ttacgatgcc attgggatat atcaacggtg gtatatccag tgattttttt
6061 ctccatttta gcttccttag ctcctgaaaa tctcgataac tcaaaaaata cgcccggtag
6121 tgatcttatt tcattatggt gaaagttgga acctcttacg tgccgatcaa cgtctcattt
6181 tcgccaaaag ttggcccagg gcttcccggt atcaacaggg acaccaggat ttatttattc
6241 tgcgaagtga tcttccgtca caggtattta ttcggcgcaa agtgcgtcgg gtgatgctgc
6301 caacttactg atttagtgta tgatggtgtt tttgaggtgc tccagtggct tctgtttcta
6361 tcagctgtcc ctcctgttca gctactgacg gggtggtgcg taacggcaaa agcaccgccg
6421 gacatcagcg ctagcggagt gtatactggc ttactatgtt ggcactgatg agggtgtcag
6481 tgaagtgctt catgtggcag gagaaaaaag gctgcaccgg tgcgtcagca gaatatgtga
6541 tacaggatat attccgcttc ctcgctcact gactcgctac gctcggtcgt tcgactgcgg
6601 cgagcggaaa tggcttacga acggggcgga gatttcctgg aagatgccag gaagatactt
6661 aacagggaag tgagagggcc gcggcaaagc cgtttttcca taggctccgc ccccctgaca
6721 agcatcacga aatctgacgc tcaaatcagt ggtggcgaaa cccgacagga ctataaagat
6781 accaggcgtt tccccctggcg gctccctcgt gcgctctcct gttcctgcct ttcggtttac
6841 cggtgtcatt ccgctgttat ggccgcgttt gtctcattcc acgcctgaca ctcagttccg
6901 ggtaggcagt tcgctccaag ctggactgta tgcacgaacc ccccgttcag tccgaccgct
6961 gcgccttatc cggtaactat cgtcttgagt ccaacccgga aagacatgca aaagcaccac
7021 tggcagcagc cactggtaat tgatttagag gagttagtct tgaagtcatg cgccggttaa
7081 ggctaaactg aaaggacaag ttttggtgac tgcgctcctc caagccagtt acctcggttc
7141 aaagagttgg tagctcagag aaccttcgaa aaaccgccct gcaaggcggt tttttcgttt
7201 tcagagcaag agattacgcg cagaccaaaa cgatctcaag aagatcatct tattaatcag
7261 ataaaatatt tctagatttc agtgcaattt atctcttcaa atgtagcacc tgaagtcagc
7321 cccatacgat ataagttgta attctcatgt tagtcatgcc ccgcgcccac cggaaggagc
7381 tgactgggtt gaaggctctc aaggg
//
```

Supplementary References

1. M. A. Moran *et al*, Sizing up metatranscriptomics. *The ISME Journal*. **7**, 237-243 (2013).
2. L. H. So *et al*, General properties of transcriptional time series in *Escherichia coli*. *Nature Genetics*. **43**, 554-560 (2011).
3. R. Lutz, H. Bujard, Independent and tight regulation of transcriptional units in *Escherichia coli* via the LacR/O, the TetR/O and AraC/I1-I2 regulatory elements. *Nucleic Acids Research*. **25**, 1203-1210 (1997).
