# Supplementary material for: Balancing a genetic toggle switch by real-time feedback control and periodic forcing
Source: Nat Commun. 2017 Nov 17;8:1671. doi: 10.1038/s41467-017-01498-0 (PMC5693866; doi:10.1038/s41467-017-01498-0)
Supplement: Supplementary file 3 — Description of Additional Supplementary Files [file 41467_2017_1498_MOESM3_ESM.pdf]

Description of Additional Supplementary Files

File Name: Supplementary Movie 1
Description: Feedback control of a cell harboring a toggle switch near its unstable equilibrium. E. coli cells, growing in a microfluidic device are observed in both GFP and RFP channels (left). Based on the ratio of the fluorescence, a PI controller adjusts in real time the concentration of aTc and IPTG (right bottom) to move and maintain a given cell (shown with an arrow on the left image, and identified as a star in the phase portrait on the right) near its unstable equilibrium point (grey point). See also Figure 2.

File Name: Supplementary Movie 2
Description: Feedback control of a cell harboring a toggle switch near its unstable equilibrium. E. coli cells, growing in a microfluidic device are observed in both GFP and RFP channels (left). Based on the ratio of the fluorescence, a Bang Bang controller adjusts in real time the concentration of aTc and IPTG (right bottom) to move and maintain a given cell (shown with an arrow on the left image, and identified as a star in the phase portrait on the right) near its unstable equilibrium point (grey point). See also Figure 3.

File Name: Supplementary Movie 3
Description: Dynamic stabilization of a population of cells. E. coli cells, growing in a microfluidic device, are observed in both GFP and RFP channels (left). The concentration of aTc and IPTG (see bottom panels) are periodically changed (120 min of 0.5 mM IPTG, 30 min of 50 ng.mL-1 aTc). All cells experience the same periodic stimulation and because of periodic forcing, they converge towards the unstable equilibrium point of the toggle switch (phase portrait, right panel). When the periodic stimulation stops, cells quickly commit to one fate. See also Figure 4.

File Name: Supplementary Movie 4
Description: Dynamic stabilization of a population of cells. E. coli cells, growing in a microfluidic device, are observed in both GFP and RFP channels (left). The concentration of aTc and IPTG (see bottom panels) are periodically changed (180 min of 0.5 mM IPTG, 30 min of 50 ng.mL-1 aTc). All cells experience the same periodic stimulation and because of periodic forcing, they converge towards the unstable equilibrium point of the toggle switch (phase portrait, right panel). When the periodic stimulation stops, cells quickly commit to either one of their two possible fates. See also Figure 4.
