# Supplementary material for: Balancing a genetic toggle switch by real-time feedback control and periodic forcing
Source: Nat Commun. 2017 Nov 17;8:1671. doi: 10.1038/s41467-017-01498-0 (PMC5693866; doi:10.1038/s41467-017-01498-0)
Supplement: Supplementary file 2 — Peer Review File [file 41467_2017_1498_MOESM2_ESM.pdf]

Reviewers' Comments:

Reviewer #1:
Remarks to the Author:
The paper claims that through the use of feedback control applied to a bistable system such as the toggle switch, a system can be dynamically maintained at an otherwise unstable equilibrium point. The novelty of the paper stems from the fact that through the use of an in silico controller, the authors able to control an unstable cellular system state stringently. Previously, others have used feedback control to increase robustness and precision of genetic circuit designs but in this paper feedback control is applied to a more complex system with an unstable equilibrium point, which is technically challenging. The paper results can help stimulate interest and demonstrate the power of using feedback control for the engineering and control of engineered living systems. The results presented in the paper are sufficient to justify the claims of the paper.

One important aspect that would be worth some additional explanation is potential applications. Although the toggle switch can be controlled using defined external control input (typically chemical inducers, e.g. IPTG and aTc) the control of natural bistable systems or network motifs embedded in larger genetic networks requires the identification of external control inputs that might not be available. Therefore, it is currently unclear how the same approach can be used practically to control other natural systems and through which external inputs. It might be worth discussing these aspects a little bit more.

Main Text
Line 69 - Reference is missing Chapter or Page Number
Line 82 – Basal concentration. What does this mean?
Line 110 – Define target (levels maybe?)
Line 118 – Numerical cell simulated cell model
Line 146 – Specify Fig. 1C. Consider including data for too fast stimulation. Maybe a graph of stabilisation against periods of stimulation

Figure 1
Line 267 – drugs. Change to chemicals or inducer chemicals
Panels C & F – axis units
Line 300-301 – Parameter units

Figure 2
Panel labelling – wrong for E: The image in E looks experimental. This is confusing as the caption says “in silico control experiment”.

Figure 3
Panels C & E – axis units

Methods
Line 352 – Explain effect and reasoning of FliA mutation
Line 368 – Include plasmid construct(s) DNA sequences in Supplementary Information document
L 457. Please provide brief technical information on how the stochastic simulations where performed. Solver/algorithm used, type of stochastic modelling (e.g. CME, SDEs), etc.
L 395. Further details on image analysis are needed. How was segmentation and tracking of individual cells was performed? Custom, commercial or other third party code?

Supplementary Information
Table S1: Include references or source of parameter values

Reviewer #2:
Remarks to the Author:
Major claims: The authors present very strong combined experimental and analytical work in which they clearly demonstrate an ability to maintain a bistable synthetic transcriptional regulatory circuit away from its stable attractors, instead keeping it near an intermediate equilibrium point that is unstable in the absence of active control (consisting of external forcing employing both proportional-integral feedback and periodic forcing).

Novelty: Closing the control loop on cellular functions using external perturbations based on real-time calculations performed outside the cell is a growing field, but I believe that the authors are correct when they state that this is the first such application to a bistable switch system.

Breadth of interest: I find the work very interesting and well done, and I believe it will be of considerable interest in the cybergenetics, synthetic biology, and biological control communities. It may have difficulty reaching a wider audience at this stage, because although connections are mentioned to the wider biological issues of cellular differentiation and decision-making (and thus to areas like controlling stem cell differentiation), this work seems to set the stage for such investigations rather than address them directly. Spending some additional time throughout the paper (especially in the introduction and discussion) emphasizing these links to wider questions could help strengthen the paper in terms of its breadth of appeal to a wide readership. My reading of the Nature Communications mandate, however, is that papers should be of interest to *specialists* in their particular field, and this work certainly does meet that criterion: it's a fascinating result, representing a novel application of control approaches to living cells. It also does lay the groundwork for future extensions into active control in a range of cellular systems, with easily foreseeable scientific and biotechnological payoffs.

Response to the reviewers.

We thank both reviewers for their very positive comments of our work and for their suggestions to improve the article.

Reviewer #1:

The paper claims that through the use of feedback control applied to a bistable system such as the toggle switch, a system can be dynamically maintained at an otherwise unstable equilibrium point. The novelty of the paper stems from the fact that through the use of an in silico controller, the authors able to control an unstable cellular system state stringently. Previously, others have used feedback control to increase robustness and precision of genetic circuit designs but in this paper feedback control is applied to a more complex system with an unstable equilibrium point, which is technically challenging. The paper results can help stimulate interest and demonstrate the power of using feedback control for the engineering and control of engineered living systems. The results presented in the paper are sufficient to justify the claims of the paper.

One important aspect that would be worth some additional explanation is potential applications. Although the toggle switch can be controlled using defined external control input (typically chemical inducers, e.g. IPTG and aTc) the control of natural bistable systems or network motifs embedded in larger genetic networks requires the identification of external control inputs that might not be available. Therefore, it is currently unclear how the same approach can be used practically to control other natural systems and through which external inputs. It might be worth discussing these aspects a little bit more.

We thank the referee for his comments. We do agree that our study is a first step towards potential applications and that at this stage it is not readily applicable to any natural systems of interest. Yet, in many cases, and notably stem cell differentiation, several external cues have been identified (*e.g.* morphogens) and it is possible to facilitate differentiation in a given fate by taking the correct culture medium. So, a number of external inputs are available and could be used to study stem cells differentiation. But more generally, modern genome editing technique such as TALEs or CRISPR/Cas9 can be used to play with gene expression. Combined with optogenetics, it may be possible to play with the level of gene expression of a given gene, at its endogenous locus and bypassing the need to use a given chemical inducer. For the sake of the genetic toggle switch it was simpler to directly use aTc and IPTG chemicals. Yet, optogenetic induction of gene expression has already been used to study signaling pathways encoding and more recently control of gene expression. We added a short discussion on this topic in the discussion section.

Other Comments:

- Line 69 - Reference is missing Chapter or Page Number

In his book, Waddington's develops the “epigenetic landscape” as a metaphor for how gene regulation modulates development. Note that here the word “epigenetics” is used in

its broadest acceptation to describe events that could not be explained by genetic principles (*i.e.* including multistability and not just chromatin modifications). Although this idea became famously illustrated in the Figure 4 of page 29 of his book, in which a marble rolls in a hilly landscape, we would like to refer more globally to his book.

- Line 82 – Basal concentration. What does this mean?

Basal concentration means the concentrations of the reference culture medium in which cells are grown and in which they display a bistable behavior. We found that having a basal level of aTc and IPTG improved the bistable behavior of our toggle switch. This is important to get a relatively symmetric bistable behavior. Indeed, the strength and leakiness of pLac and pTet are different and very difficult to predict.

- Line 110 – Define target (levels maybe?)

The sentence has been changed to: *"[…] aims to maintain the concentration of the controlled protein close to a (relatively low) target level […]"*.

- Line 118 – Numerical cell simulated cell model

We changed "*numerical cell*" to "*simulated cell*" as proposed.

- Line 146 – Specify Fig. 1C. Consider including data for too fast stimulation. Maybe a graph of stabilisation against periods of stimulation

Done. We added a supplementary figure that shows an example of periodic stimulations that are too fast and in which cells drift towards one of the attractors.

- Figure1, Line 267 – drugs. Change to chemicals or inducer chemicals

Done.

- Figure 1, Panels C & F – axis units

Done. We added the fact that fluorescence levels are measured as arbitrary units.

- Line 300-301 – Parameter units

The coefficient of the proportional term of the PI controller has no dimension, but indeed the integral term of the PI control has the dimension of the inverse of time (s^{-1}). This has been corrected.

- Figure 2, Panel labelling – wrong for E: The image in E looks experimental. This is confusing as the caption says "in silico control experiment".

The reviewer is right there was an error in the labels. The panel E is indeed showing experimental data. This has been corrected.

- Figure 3, Panels C & E – axis units.

Axis units are now indicating that fluorescence levels are measured as arbitrary units on Figure 4 panel C&E.

- Methods, Line 352 – Explain effect and reasoning of FliA mutation

To avoid cell motility we work in a background with FliA deleted. It is quite usual for bacteria and we do not foresee any effect on our study.

- Methods, Line 368 – Include plasmid construct(s) DNA sequences in Supplementary Information document

Done.

- Methods, L 457. Please provide brief technical information on how the stochastic simulations where performed. Solver/algorithm used, type of stochastic modelling (e.g. CME, SDEs), etc.

We clarified that using a stochastic interpretation of (pseudo-)reactions results in a continuous time Markov chain model and that we used the first reaction method version of the Gillespie algorithm to simulate it. We also added that propensity functions are given by the rate laws of the pseudo-reactions. The code used for the numerical study is available on our Github public lab repository (see Data availability for links and DOI)

- Methods, L 395. Further details on image analysis are needed. How was segmentation and tracking of individual cells was performed? Custom, commercial or other third party code?

We used a custom code (built in Matlab) to get fluorescence measurements from time-lapse microscopy recordings. However, as mentioned in the methods, we did not need to perform advanced segmentation cells since, for real time image analysis used for feedback loop control experiments, we only tracked at a single immobile cells that was trapped at the end of the microfluidic channels. Such cells cannot move and they did not require advanced segmentation methods.

- Supplementary Information, Table S1: Include references or source of parameter values

Appropriate references were already cited at the end of the supplementary document.

Reviewer #2:

Major claims: The authors present very strong combined experimental and analytical work in which they clearly demonstrate an ability to maintain a bistable synthetic transcriptional regulatory circuit away from its stable attractors, instead keeping it near an intermediate equilibrium point that is unstable in the absence of active control (consisting of external forcing employing both proportional-integral feedback and periodic forcing).

Novelty: Closing the control loop on cellular functions using external perturbations based on real-time calculations performed outside the cell is a growing field, but I believe that the authors are correct when they state that this is the first such application to a bistable switch system.

Breadth of interest: I find the work very interesting and well done, and I believe it will be of considerable interest in the cybergenetics, synthetic biology, and biological control communities. It may have difficulty reaching a wider audience at this stage, because

*although connections are mentioned to the wider biological issues of cellular differentiation and decision-making (and thus to areas like controlling stem cell differentiation), this work seems to set the stage for such investigations rather than address them directly. Spending some additional time throughout the paper (especially in the introduction and discussion) emphasizing these links to wider questions could help strengthen the paper in terms of its breadth of appeal to a wide readership. My reading of the Nature Communications mandate, however, is that papers should be of interest to *specialists* in their particular field, and this work certainly does meet that criterion: it's a fascinating result, representing a novel application of control approaches to living cells. It also does lay the groundwork for future extensions into active control in a range of cellular systems, with easily foreseeable scientific and biotechnological payoffs.*

We thank the referee for his comments. The reviewer is correct that "*this work seems to set the stage for such investigations rather than address them directly*". It's hard at this stage to be more specific on the potential applications (see our answer to reviewer#1). But we can anticipate that dynamic, real time control of cellular unstable state could be used to study and play with stem cell differentiation, for example by adjusting dynamically the concentrations of growth factors and morphogens which are known to induce or inhibit specific cell fate.
